# Suppression of histone deacetylases by SAHA relieves bone cancer pain in rats via inhibiting activation of glial cells in spinal dorsal horn and dorsal root ganglia

**DOI:** 10.1186/s12974-020-01740-5

**Published:** 2020-04-22

**Authors:** Xiao-Tao He, Xiao-Fan Hu, Chao Zhu, Kai-Xiang Zhou, Wen-Jun Zhao, Chen Zhang, Xiao Han, Chang-Le Wu, Yan-Yan Wei, Wei Wang, Jian-Ping Deng, Fa-Ming Chen, Ze-Xu Gu, Yu-Lin Dong

**Affiliations:** 1 Department of Human Anatomy, Histology and Embryology & K.K. Leung Brain Research Centre, Preclinical School of Medicine, The Fourth Military Medical University, Xi’an, 710032 People’s Republic of China; 2 Department of Periodontology, School of Stomatology, The Fourth Military Medical University, Xi’an, 710032 People’s Republic of China; 3 Department of Orthopedics, Xijing Hospital, The Fourth Military Medical University, Xi’an, 710032 People’s Republic of China; 4 Department of Spine Surgery, Renji Hospital, School of Medicine, Shanghai Jiao Tong University, Shanghai, 200127 People’s Republic of China; 5 Student Brigade, The Fourth Military Medical University, Xi’an, 710032 People’s Republic of China; 6 State Key Laboratory of Military Stomatology, Department of Anesthesiology, School of Stomatology, The Fourth Military Medical University, Xi’an, 710032 People’s Republic of China; 7 Department of Neurosurgery, Tangdu Hospital, The Fourth Military Medical University, Xi’an, 710032 People’s Republic of China; 8 State Key Laboratory of Military Stomatology, Department of Orthodontics, School of Stomatology, The Fourth Military Medical University, Xi’an, 710032 People’s Republic of China

**Keywords:** Bone cancer pain, HDACs, Glial cells, Neuroinflammation, Spinal dorsal horn

## Abstract

**Background:**

Robust activation of glial cells has been reported to occur particularly during the pathogenesis of bone cancer pain (BCP). Researchers from our group and others have shown that histone deacetylases (HDACs) play a significant role in modulating glia-mediated immune responses; however, it still remains unclear whether HDACs are involved in the activation of glial cells during the development of BCP.

**Methods:**

BCP model was established by intra-tibia tumor cell inoculation (TCI). The expression levels and distribution sites of histone deacetylases (HDACs) in the spinal dorsal horn and dorsal root ganglia were evaluated by Western blot and immunofluorescent staining, respectively. Suberoylanilide hydroxamic acid (SAHA), a clinically used HDAC inhibitor, was then intraperitoneally and intrathecally injected to rescue the increased expression levels of HDAC1 and HDAC2. The analgesic effects of SAHA administration on BCP were then evaluated by measuring the paw withdrawal thresholds (PWTs). The effects of SAHA on activation of glial cells and expression of proinflammatory cytokines (TNF-α, IL-1β, and IL-6) in the spinal dorsal horn and dorsal root ganglia of TCI rats were further evaluated by immunofluorescent staining and Western blot analysis. Subsequently, the effects of SAHA administration on tumor growth and cancer cell-induced bone destruction were analyzed by hematoxylin and eosin (HE) staining and micro-CT scanning.

**Results:**

TCI caused rapid and long-lasting increased expression of HDAC1/HDAC2 in glial cells of the spinal dorsal horn and dorsal root ganglia. Inhibiting HDACs by SAHA not only reversed TCI-induced upregulation of HDACs but also inhibited the activation of glial cells in the spinal dorsal horn and dorsal root ganglia, and relieved TCI-induced mechanical allodynia. Further, we found that SAHA administration could not prevent cancer infiltration or bone destruction in the tibia, which indicated that the analgesic effects of SAHA were not due to its anti-tumor effects. Moreover, we found that SAHA administration could inhibit GSK3β activity in the spinal dorsal horn and dorsal root ganglia, which might contributed to the relief of BCP.

**Conclusion:**

Our findings suggest that HDAC1 and HDAC2 are involved in the glia-mediated neuroinflammation in the spinal dorsal horn and dorsal root ganglia underlying the pathogenesis of BCP, which indicated that inhibiting HDACs by SAHA might be a potential strategy for pain relief of BCP.

## Background

Cancer is one of the major types of disease that seriously threatens public health. Given the current trend of an increase in survival rates and survival time of patients with advanced cancer, it is predicted that there will be 22.2 million new cancer cases and 13.2 million cancer-related deaths in 2030 [1]. Many common cancers, such as those arising from the breast, prostate, kidney, and lung, avidly metastasize to the skeleton, which induces severe bone pain [2]. Due to the lack of elucidation of its mechanism, bone cancer pain (BCP) remains one of the most intractable pains to be fully control. Clinically, systematic administration of morphine is a major option for treating BCP [3]. However, due to the significant opioid-related side effects (e.g., constipation and analgesic tolerance), it is critically urgent to elucidate the mechanism underlying BCP and search for efficacious analgesic drugs with fewer side effects to improve patients’ quality of life.

Histone deacetylases (HDACs) have been reported to be closely correlated with the aberrant transcriptional responses in the periphery and central nervous system during chronic pain [4–6]. Series of studies have shown HDAC inhibitors (HDACi) can exert antinociceptive effects on inflammatory and neuropathic pain via modulating synaptic plasticity of neuronal cells [7–9]. However, the neurochemical changes in BCP are quite different from non-cancer pain, such as inflammatory and neuropathic pain [10, 11]. It has been reported that remarkable and sustainable activation of glial cells particularly occurs in the spinal dorsal horn and peripheral nervous system during BCP [10, 12, 13]. The over-activated astrocytes and microglia can release a variety of proinflammatory cytokines (e.g., TNF-α, IL-1β and IL-6) to trigger persistent pain [14, 15]. Currently, mounting evidence has shown that HDACs could epigenetically regulate gene expression and inflammatory responses of glial cells in models of systemic immune activation [16, 17]. Several studies have demonstrated that inhibition of HDACs can exhibit immunosuppressive effects on microglia-mediated neuroinflammation based on a direct impairment of the transcriptional machinery [16, 18, 19]. Thus, we hypothesized that inhibiting HDACs might exert analgesic effects on BCP through suppressing glia-mediated neuroinflammation, and HDACi could relieve BCP by inhibiting HDACs in spinal dorsal horn and dorsal root ganglia.

To test our hypothesis, BCP models were established via intra-tibia tumor cell inoculation (TCI). Then we compared the distribution sites of HDACs (HDAC1 and HDAC2) in the spinal dorsal horn and dorsal root ganglia during BCP and neuropathic pain induced by spinal nerve ligation (SNL) to evaluate the different roles of HDACs in BCP and SNL. Subsequently, suberoylanilide hydroxamic acid (SAHA), a type of HDACi clinically approved by the Food and Drug Administration, was intraperitoneally injected to explore whether inhibiting HDACs could sufficiently downregulate the activation of glial cells to alleviate BCP. Given that glycogen synthase kinase-3 beta (GSK3β) was a key point of convergence of many signaling pathways to modify neuroinflammation, we further explored whether inhibiting HDACs could inhibit GSK3β activities to alleviate BCP.

## Methods

### Animals, anesthesia, drugs, and drug administration

Female Sprague-Dawley rats (180–200 g) were provided by the Laboratory Animal Center of the Fourth Military Medical University (FMMU). Five to six adult female rats were housed per cage under specific pathogen-free conditions with soft bedding under a controlled temperature (22 ± 2 °C). Before the experiments, all animals were adapted to the experimental circumstances for 5–7 days. All surgeries were performed under anesthesia with sodium pentobarbital (50 mg/kg, i.p.). Animals were randomly divided into fourteen groups. For each group of experiments, the animals were age- and body weight-matched. SAHA and AR-A014418 were purchased from Selleckchem (Houston, TX, USA). The drug doses were selected based on previous reports [20–22] and our preliminary experiments. SAHA was dissolved in dimethyl sulfoxide (DMSO, Sigma, St. Louis, MO, USA) and diluted in physiological saline to a final concentration of 5% DMSO (v/v); AR-A014418 was dissolved in DMSO and then was diluted in physiological saline to a final concentration of 0.4% DMSO. For i.p. injection, SAHA (50 mg/kg) or AR-A014418 (3 mg/kg) were injected once daily for 21 consecutive days to evaluate the analgesic effects of SAHA and AR-A014418 on BCP. For i.t. injection, SAHA (250 μg/kg, 10 μL in volume) or AR-A014418 (400 ng/kg, 10 μL in volume) were injected once daily for 14 consecutive days and the doses of drug administration were according to previously reported researches [21, 22]. DMSO (5%) was used as the vehicle treatment for SAHA treatment. Similarly, the same amount and concentration of vehicle was used as the vehicle treatment for AR-A014418 treatment. Rats with i.p. or i.t. administration were sacrificed 21 or 14 days after the operations for immunofluorescent staining or protein determinations, respectively.

### Cell preparation

Walker 256 rat mammary gland carcinoma cells were purchased from American Type Culture Collection (ATCC, USA). As described previously [23, 24], 0.5 mL (2 × 10^7^ cells/mL) cancer cells were injected into the abdominal cavities of female rats. Seven to ten days later, 2 mL of extracted ascitic fluid was centrifuged at 1500 rpm for 3 min. Then the pellet was washed with phosphate-buffered saline (PBS) and resuspended in 1 mL of PBS. Subsequently, the cells were diluted with PBS to achieve a final concentration of 5 × 10^7^ cells/mL. The cell suspension was kept on ice until intra-tibia injection. In the Sham group, the same procedures were followed, except that heat-killed carcinoma cell suspension with equal volume and density was administered instead of normal carcinoma cells.

### BCP model

As described in previously reported procedure [23, 24], after complete anesthesia, the skin of the right tibia of the rat was cut, and 10 μL of the Walker 256 carcinoma cell suspension (5 × 10^5^ cells) was slowly injected into the intramedullary cavity of the right tibia. The injection site was immediately sealed using bone wax when the syringe was removed. In the Sham group, the same procedures were followed, except that an equal volume of heat-treated carcinoma cells was administered instead of normal carcinoma cells. Sham rats on postoperative day (POD) 7 were used as the control for Western blot and immunofluorescent staining.

### Spinal nerve ligation models

Six rats were anesthetized and placed in a prone position. Then, the left L6 transverse process was carefully removed with a small rongeur to expose the L4 and L5 spinal nerves. The L5 spinal nerve was then carefully isolated and tightly ligated with a 6–0 silk thread 2–5 mm distal to the dorsal root ganglia. Finally, the wound and surrounding skin were sutured. Animals showing obvious mechanical allodynia were used for the subsequent immunofluorescent staining. The surgical procedures for the Sham group were identical to those of the model group, except that the spinal nerves were not ligated. Sham rats on POD 7 were used as the control for immunofluorescent staining.

### Intrathecal implantation

The intrathecal implantation was performed as described in our previously published study [24]. Briefly, an intrathecal PE-10 catheter (Becton Dickinson, San Jose, CA, United States) was placed intrathecally in the lumbar enlargement under complete anesthesia. To confirm the success of catheterization, 10 μL of lidocaine (2%) was injected through the catheter on the next day. Rats showing immediate hind limb paralysis after injection were prepared for the TCI. At the end of each experiment, the position of the polyethylene tubing in the intrathecal space was visually verified by exposing the lumbar spinal cord. Data from rats with incorrect PE tubing position were discarded from the study.

### Nociceptive behavioral test

The rats were habituated to the testing environment for 5–7 days before baseline testing. Baseline nociceptive tests were carried out for three consecutive days prior to the TCI. Animals were discarded in the present study when the difference of the baseline before and after surgery was greater than 4 g. In the test, each rat was put individually under inverted plastic boxes (30 × 30 × 50 cm^3^) on an elevated mesh floor. After 30 min of acclimation, mechanical thresholds were tested using von Frey filaments (Stoelting, Kiel, WI, USA) by experimenters who were blinded with respect to the group assignments. The ipsilateral hind paws were pressed upwards using Von Frey filaments (Stoelting, Kiel, WI, United States) with gradually increasing stiffness (0.4, 0.6, 1.0, 2, 4, 6, 8, 10, and 15 g) to cause a slight bend and left for 5–6 s. Each filament was applied ten times, and the minimal value that caused at least six responses was recorded as the paw withdraw threshold (PWT). Acute withdrawal, biting, licking, or shaking of the ipsilateral hind limb and vocalization were considered as positive signs of withdrawal.

### Radiological analysis

To confirm TCI-induced bone destruction, the tibia of rats were radiographed on POD 21. They were placed supine on an X-ray film (Henry Schein blue sensitive film, Henry Schein, NY, USA) after analgesia and were exposed to an X-ray source (Emerald 125) for 1/20 s at 40 KVP. The X-ray film was developed by a film developer (Konica SRX-101A, Konica Minolta, Tokyo, Japan).

### Bone histology

On POD 21, rats were anesthetized with an overdose of pentobarbital (60 mg/kg, i.p.) and were perfused with 150 mL of 0.9% normal saline. The tibial bones ipsilateral to the TCI were then removed and decalcified in 10% ethylenediaminetetraacetic acid for 3 weeks. The bones were rinsed, dehydrated, and then embedded in paraffin, cut into 7-μm cross-sections using a rotary microtome (Reichert-Jung 820, Cambridge Instruments GmbH, Nussloch, Germany), and stained with hematoxylin and eosin (HE) to visualize the extent of tumor infiltration and bone destruction. Finally, the stained specimens were observed under a bright-field microscope.

### MicroCT analysis

The tibia bone was carefully harvested and fixed in 4% paraformaldehyde until they were scanned with a high-resolution microCT (GE healthcare, Madison, WI, USA). The scans were performed in the long axis of the diaphysis, with the following basic scan parameters: voltage, 80 kVp; current, 80 μA; exposure time, 3000 ms; total rotation angle, 360°; and rotation angle of increment, 0.4°. Analyses were performed using the Micview V2.1.2 software. The 3D datasets were low-pass filtered and segmented with a fixed threshold filter (1000 mg HA/cm^3^) according to the current guidelines [25]. For quantitative analysis of bone destruction, the volume of interest was defined as a round-shaped yellow region that started at a distance of 0.1 mm of the top end of the growth plate and extended to the proximal end of the tibia with a distance of 1.5 mm. Only spongiosa was included in the volume of interest. The bone mineral density of the trabeculae was then calculated based on the microCT scanning.

### Protein determination

According to previous descriptions [15, 23], the L4–5 spinal cord segment was dissected on dry ice according to the termination of the L4 and L5 dorsal ganglia roots. Then, the spinal segment was cut into left and right halves from the midline. The right half was further split into the dorsal and ventral horns at the level of the central canal. The L4 and L5 dorsal root ganglia were obtained according to the methods reported previously [24]. The total protein of the harvested specimens was extracted using lysis buffer (Beyotime, Shanghai, P.R. China) with a mixture of proteinase and phosphatase inhibitors (Sigma). The total protein contents of the samples were then equalized using the bicinchoninic acid method (with reagents from Beyotime). The electrophoresis samples were denatured at 100 °C for 5 min and loaded onto 10% SDS–polyacrylamide gels with standard Laemmli solutions (Bio-Rad Laboratories, CA, USA). The proteins were then electroblotted onto a polyvinylidene difluoride membrane (PVDF; Millipore, Billerica, MA, USA). Subsequently, the membranes were incubated in Tris-buffered saline containing 0.02% Tween-20 (TBS-T) and 4% non-fat milk for 1 h and incubated overnight under gentle agitation with primary antibodies. The following primary antibodies were used: mouse anti-HDAC1 IgG (1:1000; Cell Signaling Technology, Beverly, MA, USA), rabbit anti-HDAC2 IgG (1:1000; Cell Signaling Technology), mouse anti-HDAC3 IgG (1:1000; Cell Signaling Technology), rabbit anti-HDAC4 IgG (1:1000; Cell Signaling Technology), rabbit anti-HDAC5 IgG (1:1000; Cell Signaling Technology), rabbit anti-HDAC6 IgG (1:1000; Cell Signaling Technology), mouse anti-glial fibrillary acidic protein IgG (GFAP, a marker of astrocytes, 1:5000, Chemicon, Temecula, CA, USA), goat anti-ionized calcium-binding adaptor molecule 1 IgG (Iba-1, a marker of microglia, 1:1000, Abcam, Cambridge, MA, USA), goat anti-tumor necrosis factor-α IgG (TNF-α, 1:300, Santa Cruz Biotechnology, Santa Cruz, CA, USA), rabbit anti-interleukin (IL)-1β IgG (1:300; Santa Cruz Biotechnology), goat anti-IL-6 IgG (1:300; Santa Cruz Biotechnology), rabbit anti-p-GSK3β IgG (1:1000; Cell Signaling Technology), mouse anti-GSK3β IgG (1:1000; Cell Signaling Technology), and mouse-anti-β-actin IgG (1:3000, ComWin Biotech, Beijing, P.R. China). Horseradish peroxidase-conjugated anti-rabbit, anti-mouse, or anti-goat antibody IgG (1: 5000; Amersham Pharmacia Biotech, Piscataway, NJ, USA) was used as the secondary antibodies. The membranes were then detected using enhanced chemiluminescence (ECL) kits (Amersham Life Science, Amersham, UK). In addition, the data were analyzed using a Molecular Imager (ChemiDoc XRS; Bio-Rad) and the associated software ImageJ Plus software (National Institute of Health, Maryland, USA).

### Quantitative real-time polymerase chain reaction

Real-time reverse transcriptional polymerase chain reaction was performed as previously described [26]. The primers employed in the current study are shown in Additional file 4: Table S1. The housekeeping gene *glyceraldehyde 3*-*phosphate dehydrogenase* was used for normalization.

### Immunofluorescence staining

Rats were deeply anesthetized, and then perfused with 100 mL of 0.9% saline followed by 500 mL of 0.1 M phosphate buffer (pH 7.3) containing 4% paraformaldehyde and 2% picric acid. After perfusion, the L4–5 spinal segment and dorsal root ganglia (L4 or L5) were immediately removed and then cryoprotected for 24 h at 4 °C in 0.1 M phosphate buffer containing 30% sucrose. Transverse frozen spinal sections (25 μm in thickness) were then cut with a cryostat (Leica CM1800; Heidelberg, Germany) and collected serially into several dishes.

The sections were incubated for 1 h at room temperature and overnight at 4 °C with primary antibodies (shown in Table 1) diluted in 0.01 M PBS containing 0.3% (v/v) Triton X-100, 0.25% (w/v) λ-carrageenan, and 5% (v/v) donkey serum (PBS-XCD). For double immunofluorescence, sections were incubated with a mixture of two primary antibodies followed by a mixture of the two respective secondary antibodies (shown in Table 1). Between the two adjacent steps, the sections were thoroughly rinsed with 0.01 M PBS. Confocal images were obtained using a confocal laser microscope (FV-1000; Olympus, Tokyo, Japan) with the appropriate laser beams and filter settings for Alexa 488 (excitation, 488 nm; emission, 510–530 nm) and Alexa 594 (excitation, 543 nm; emission, 590–615 nm), and digital images were captured with a FluoView 1000 microscope (Olympus). The specificity of the staining was tested on the sections in the second dish by omission of the primary specific antibodies. No immunoreactive products were detected (data not shown).

**Table 1:** Antibodies used in immunofluorescent staining

	Antigens	Primary antisera	Secondary antisera
Single staining	HDAC1 HDAC2 GFAP Iba-1	Rabbit anti-HDAC1 IgG (1: 200; Sigma) Rabbit anti-HDAC2 IgG (1:400; Cell Signaling Technology) Mouse anti-GFAP IgG (1:5000; Chemicon) Goat anti-Iba-1 IgG (1:800; Abcam)	Alexa 488 donkey anti-rabbit IgG (1: 500) (Invitrogen) Alexa 594 donkey anti-mouse IgG (1: 500) (Invitrogen) Alex 594 donkey anti-goat IgG (1: 500) (Invitrogen)
Double staining	HDAC1/NeuN HDAC1/GFAP HDAC1/Iba-1 HDAC2/NeuN HDAC2/GFAP HDAC2/Iba-1	Rabbit anti-HDAC1 IgG (1: 200; Sigma) Rabbit anti-HDAC2 IgG (1:400; Cell Signaling Technology) Mouse anti-NeuN IgG (1: 2000; Chemicon) Mouse anti-GFAP IgG (1:5000; Chemicon) Goat anti-Iba-1 IgG (1:800; Abcam)	

### Statistical analysis

All data were collected by researchers who were blinded to the surgeries and reagents used. GraphPad Prism version 5.01 for Windows (San Diego, CA, USA) was used to conduct all statistical analyses. Nociceptive behavioral tests over time among groups were tested with two-way repeated-measures analysis of variance (ANOVA) followed by Bonferroni’s post hoc tests. Differences in Western blot values over time for each group were tested using one-way ANOVA with a Student-Newman-Keuls (SNK) post hoc test. All data are presented as the mean ± SEM. The criterion for statistical significance was a *p* value less than 0.05.

## Results

### TCI-induced bone destruction and mechanical allodynia

**Fig. 1:**
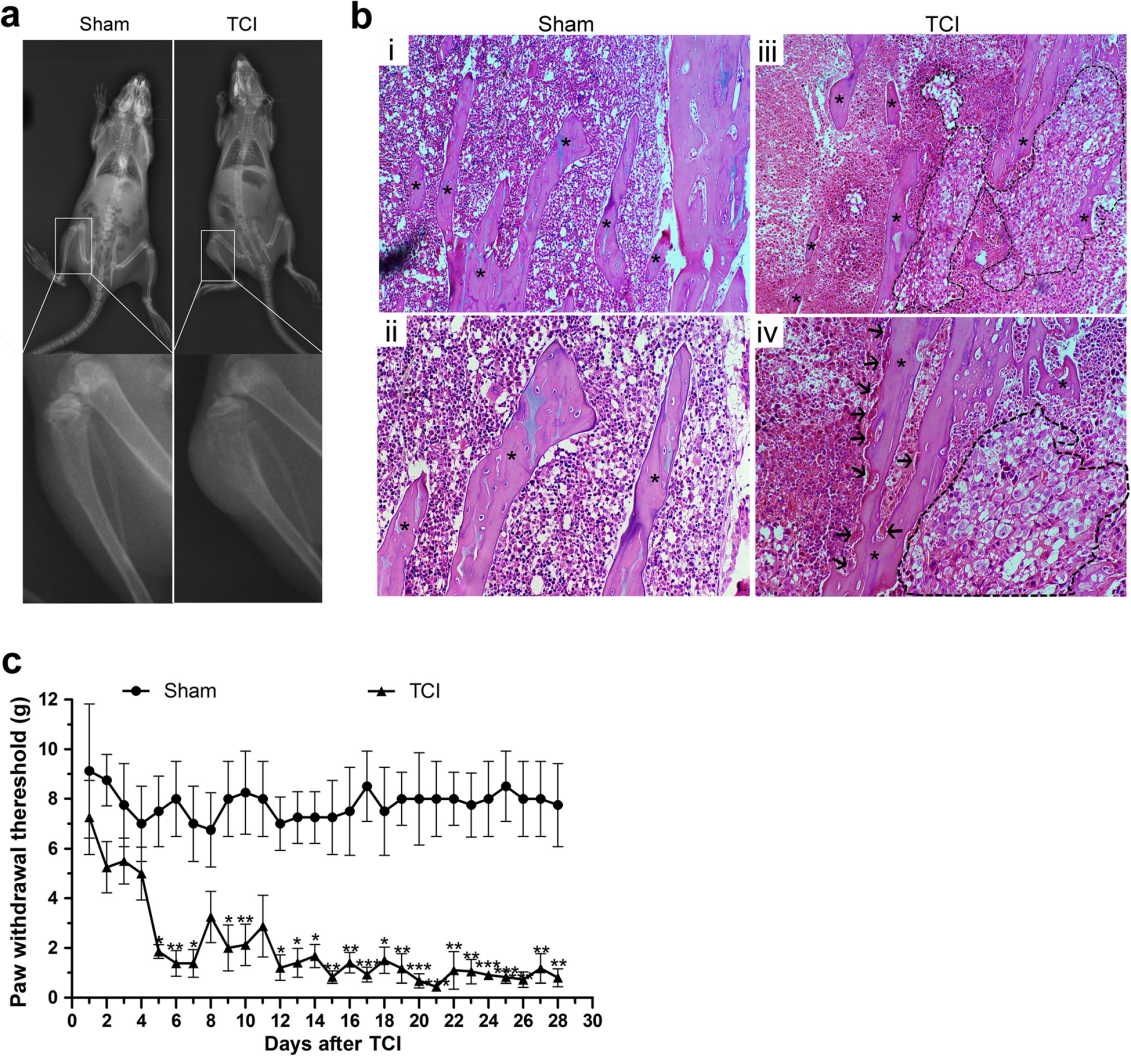
TCI-induced bone destruction and mechanical allodynia. **a** Radiographs of the tibia bone in the Sham and TCI rats on POD 21. **b** HE staining of the trabecular bone in the Sham and the TCI group on POD 21. **b** (i, ii) Representative images of HE staining showed regular arrangement of trabecular bone (asterisks) in tibial marrow cavity of the Sham group. **b** (iii, iv) Representative images of HE staining showed cancer cells (within the dotted lines) and osteoclastic resorption pits (arrows) on trabecular surface in tibial marrow cavity of the TCI group on POD 21. Original magnification: 100 (top row), 200 (bottom row). **c** TCI-induced prominent mechanical allodynia from POD 5 to POD 28 (*n* = 8). Data are expressed as the mean ± SEM. *p < 0.05, **p < 0.01, ***p < 0.001 versus the Sham group

X-ray radiograph showed that there were visibly radiolucent lesions in the proximal epiphysis of the tibias in the TCI group as compared with Sham group on POD 21 (Fig. 1a). HE staining showed obvious cancer cell infiltration (within the dotted lines) and osteoclastic resorption pits (black arrows) attaching to trabecular surfaces in tibial marrow cavity of TCI rats (Fig. 1b(iii, iv)). In contrast, neither cancer cells nor osteoclasts were observed in the tibial marrow cavity of the Sham rats (Fig. 1b(i, ii)).

Compared with the relatively stable PWTs in the Sham group, TCI induced a progressive and dramatic reduction in the PWTs of the ipsilateral hind paws with regard to Von Frey hairs stimulation. The mechanical allodynia tests showed that the PWTs of the TCI rats were dramatically decreased on POD 5, and maintained at a significantly lower threshold compared with that of the Sham group till POD 28 (Fig. 1c).

### TCI-induced upregulation of HDAC1 and HDAC2 in the spinal dorsal horn

**Fig. 2:**
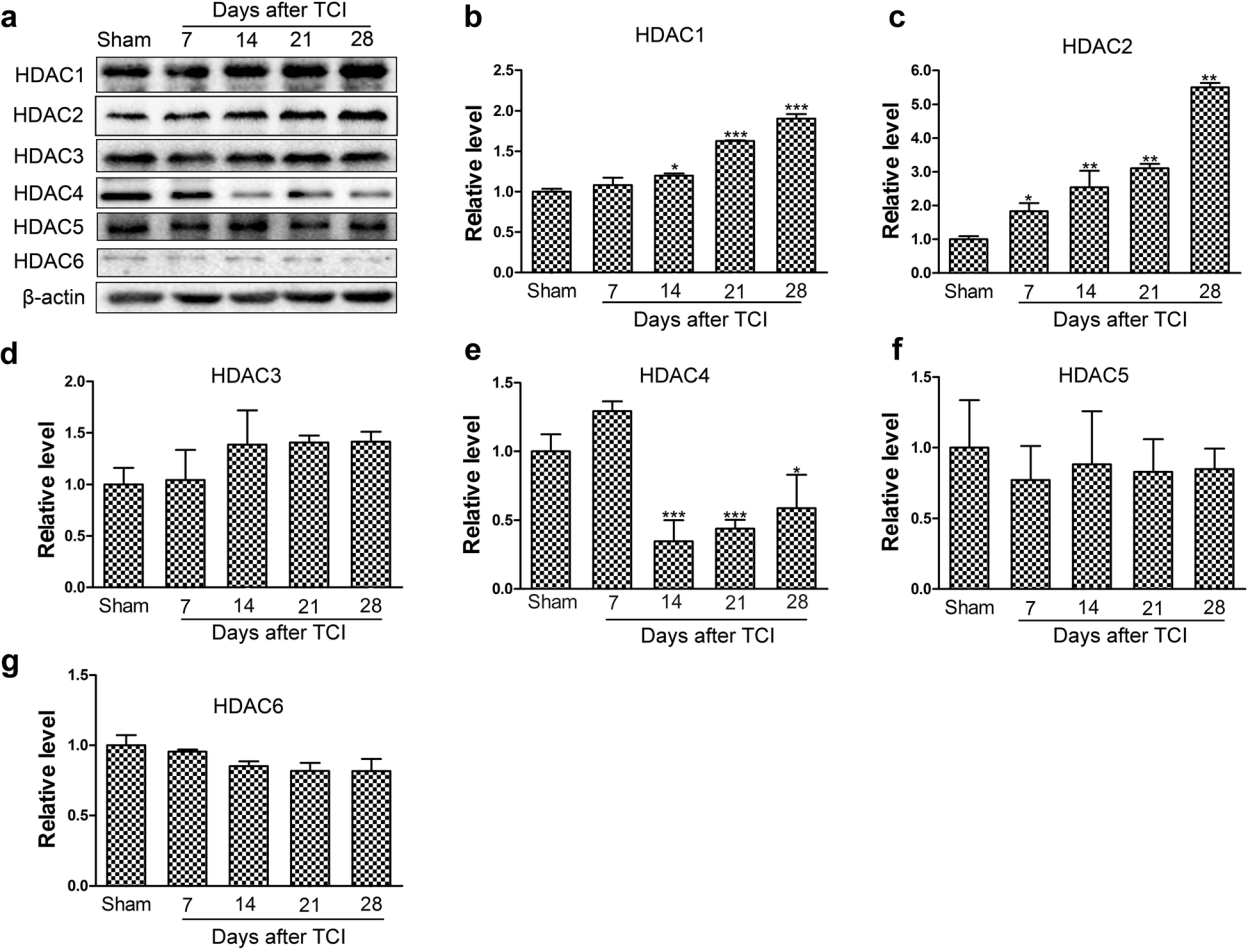
TCI-induced time-dependent changes of HDAC1~HDAC6 expression in the spinal dorsal horn at various time points (Sham, POD 7, POD 14, POD 21 and POD 28). Representative bands (**a**) and quantitative analysis of HDAC1~HDAC6 (**b**–**g**) in the spinal dorsal horn at various time points following TCI (*n* = 4). Analysis was based on the mean gray values and normalized to β-actin. Data are expressed as the mean ± SEM. *p < 0.05, **p < 0.01, ***p < 0.001 versus the Sham group

To explore the engagement of HDACs in central sensitization during pathogenesis of BCP, we examined the expression levels of HDAC1~HDAC6 in the spinal dorsal horn ipsilateral to the TCI at various time points (Sham, POD 7, POD 14, POD 21 and POD 28). Western blot analysis showed that TCI induced continuous and significant increase in the expression levels of HDAC1 in the spinal dorsal horn from POD 14 to POD 28 (Fig. 2a, b). The expression levels of HDAC2 also increased rapidly and significantly from POD 7 to POD 28 following TCI (Fig. 2c). In contrast, the expression levels of HDAC4 decreased continually and significantly from POD 14 to POD 28 (Fig. 2e). However, the expression levels of HDAC3, HDAC5 and HDAC6 proteins maintained unchanged following TCI (Fig. 2d, f–g).

Consistent with Western blot analysis, the quantitative real-time polymerase chain reaction (qRT-PCR) results showed that the mRNA expression levels of *HDAC1* and *HDAC2* in the spinal dorsal horn increased persistently following TCI (Additional file 1: Figure S1a, b). Conversely, the mRNA expression levels of HDAC4 continually decreased following TCI, and a significant difference was observed on POD 14 (Additional file 1: Figure S1d). However, the mRNA expression levels of *HDAC3*, *HDAC5*, and *HDAC6* in the spinal dorsal horn did not change obviously following TCI (Additional file 1: Figure S1c, e and f).

### TCI-induced upregulation of HDAC1 in the spinal dorsal horn was mainly located in neuron and astrocytes

**Fig. 3:**
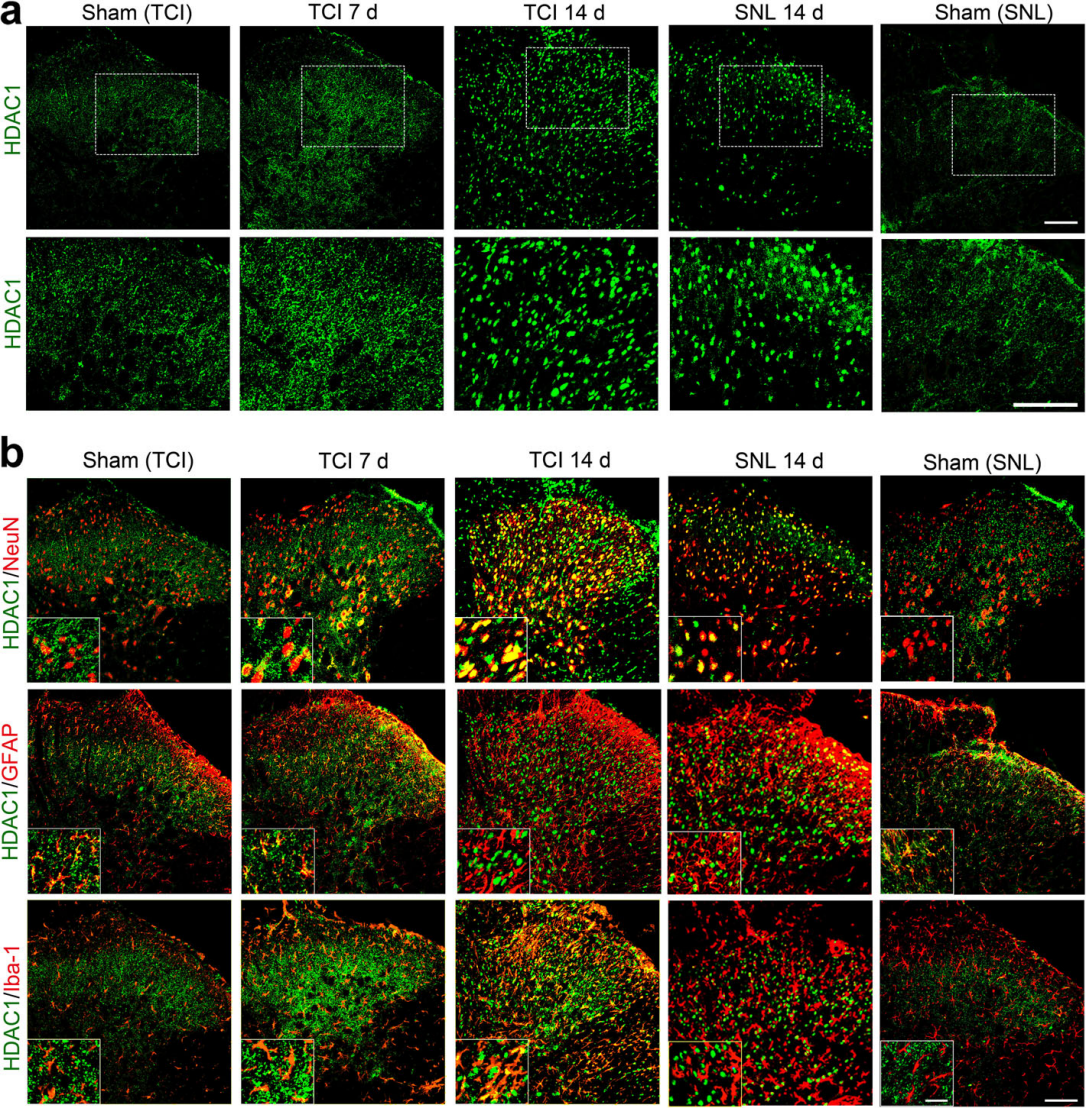
TCI-induced upregulation of HDAC1 in the spinal dorsal horn following TCI or SNL. **a** Immunofluorescent staining of HDAC1 in the spinal dorsal horn at various time points (Sham, POD 7 and POD 14 for TCI; Sham and POD 14 for SNL). Scale bar = 100 μm. **b** Double immunofluorescent staining showing the co-localization of HDAC1 (green) with neurons (NeuN, red), astrocytes (GFAP, red), and microglia (Iba-1, red) at various time points (Sham, POD 7 and POD 14 for TCI; Sham and POD 14 for SNL). Scale bar = 100 μm (outside); 50 μm (inside)

To explore the roles of HDAC1 and HDAC2 in the spinal dorsal horn during BCP, we further investigated the expression and distribution of HDAC1 and HDAC2 at various time points (Sham, POD 7, and POD 14) following TCI. Rats with SNL (Sham and POD 14) were included in the present study to identify different roles of HDACs in rat models of BCP and neuropathic pain. Immunofluorescent staining showed that the distributions of HDAC1-like immunoreactivities (green fluorescence) were observed in the spinal dorsal horn. Following either TCI or SNL, the immunofluorescent intensity of HDAC1 in the spinal dorsal horn was markedly increased (Fig. 3a). Double immunofluorescent staining showed that HDAC1 staining was mainly expressed in astrocytes (GFAP, red) in the spinal dorsal horn of the sham-operated rats for TCI or SNL. However, spinal HDAC1 in microglia and neurons was sharply increased on POD 7 and POD 14 following TCI, and only a few HDAC1 was located in astrocytes on POD 14. In contrast, the increased HDAC1 following SNL was only observed in neuronal cells (Fig. 3b).

### TCI-induced upregulation of HDAC2 in spinal dorsal horn was mainly located in astrocytes

**Fig. 4:**
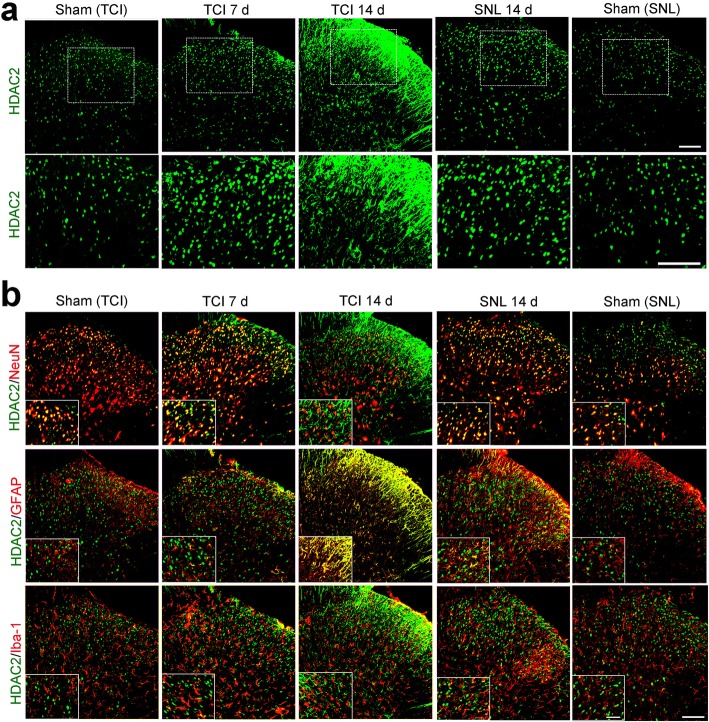
TCI-induced upregulation of HDAC2 in the spinal dorsal horn following TCI or SNL. **a** Immunofluorescent staining of HDAC2 in the spinal dorsal horn at various time points (Sham, POD 7 and POD 14 for TCI; Sham and POD 14 for SNL). Scale bar = 100 μm. **b** Double immunofluorescent staining showing the co-localization of HDAC2 (green) with neurons (NeuN, red), astrocytes (GFAP, red), and microglia (Iba-1, red) at various time points (Sham, POD 7 and POD 14 for TCI; Sham and POD 14 for SNL). Scale bar = 100 μm (outside); 50 μm (inside)

Immunofluorescent staining showed that the distributions of HDAC2-like immunoreactivities (green fluorescence) were observed in the spinal dorsal horn. In accordance with the Western blot results, confocal images showed that the immunofluorescent intensity of HDAC2 in the spinal dorsal horn markedly increased following TCI or SNL (Fig. 4a). Double immunofluorescent staining showed that HDAC2 was mainly expressed in neurons (NeuN, red) in the spinal dorsal horn of sham-operated rats for TCI or SNL. However, the co-localization of HDAC2 (green fluorescence) and GFAP (red fluorescence) increased sharply on POD 7 and POD 14 following TCI. In contrast, the increased HDAC2 following SNL was mainly located in neuronal cells on POD 14, although HDAC2 was also observed in astrocytes or microglia (Fig. 4b).

### The expression levels of HDAC1 and HDAC2 in satellite glia cells of the dorsal root ganglia also increased following TCI

**Fig. 5:**
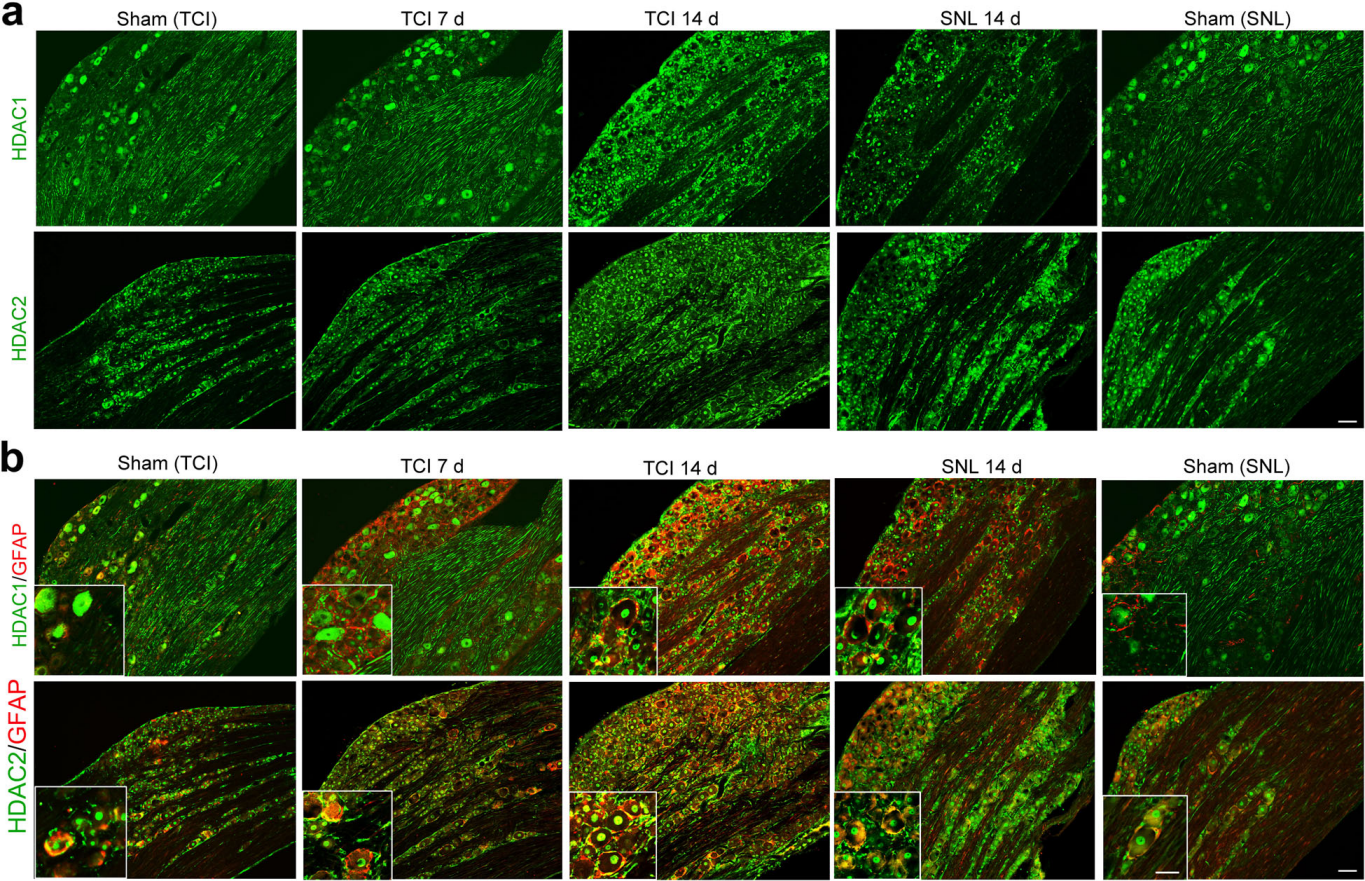
TCI-induced upregulation of HDAC1 and HDAC2 in the dorsal root ganglia following TCI or SNL. **a** Immunofluorescent staining of HDAC1 and HDAC2 in the dorsal root ganglia at various time points (Sham, POD 7 and POD 14 for TCI; Sham and POD 14 for SNL). Scale bar = 100 μm. **b** Double immunofluorescent staining showing the co-localization of HDAC1/HDAC2 (green) and satellite glial cells (GFAP, red) at various time points (Sham, POD 7, and POD 14 for TCI; Sham and POD 14 for SNL). Scale bar = 100 μm (outside); 50 μm (inside)

Similarly, immunofluorescent staining showed that the immunofluorescent intensities of HDAC1 in the dorsal root ganglia increased following both SNL and TCI (Fig. 5a). The double immunofluorescent staining showed that there was no co-localization of HDAC1 (green fluorescence) and GFAP (activated marker of satellite glial cells, red fluorescence) in the dorsal root ganglia of sham-operated rats for TCI or SNL. However, the co-localization of HDAC1 and GFAP increased on POD 7 and POD 14 following both TCI and SNL (Fig. 5b). In addition, the immunofluorescent staining showed that HDAC1-like immunoreactivities were mainly located in the cytoplasm of cells in the dorsal root ganglia of Sham rats for TCI and SNL, while located in the nuclei of cells in the TCI and SNL groups (Fig. 5a, b).

As for HDAC2, immunofluorescent staining also showed that the immunofluorescent intensities of HDAC2 in the dorsal root ganglia increased following SNL and TCI (Fig. 5a). The double immunofluorescent staining exhibited some co-localization of HDAC2 and GFAP in the dorsal root ganglia of sham-operated rats for TCI and SNL. However, the co-localization of HDAC2 (green fluorescence) and GFAP (red fluorescence) increased on POD 7 and POD 14 following both TCI and SNL (Fig. 5b).

### The effects of SAHA on TCI-induced mechanical allodynia and glia-mediated neuroinflammation in the spinal dorsal horn

**Fig. 6:**
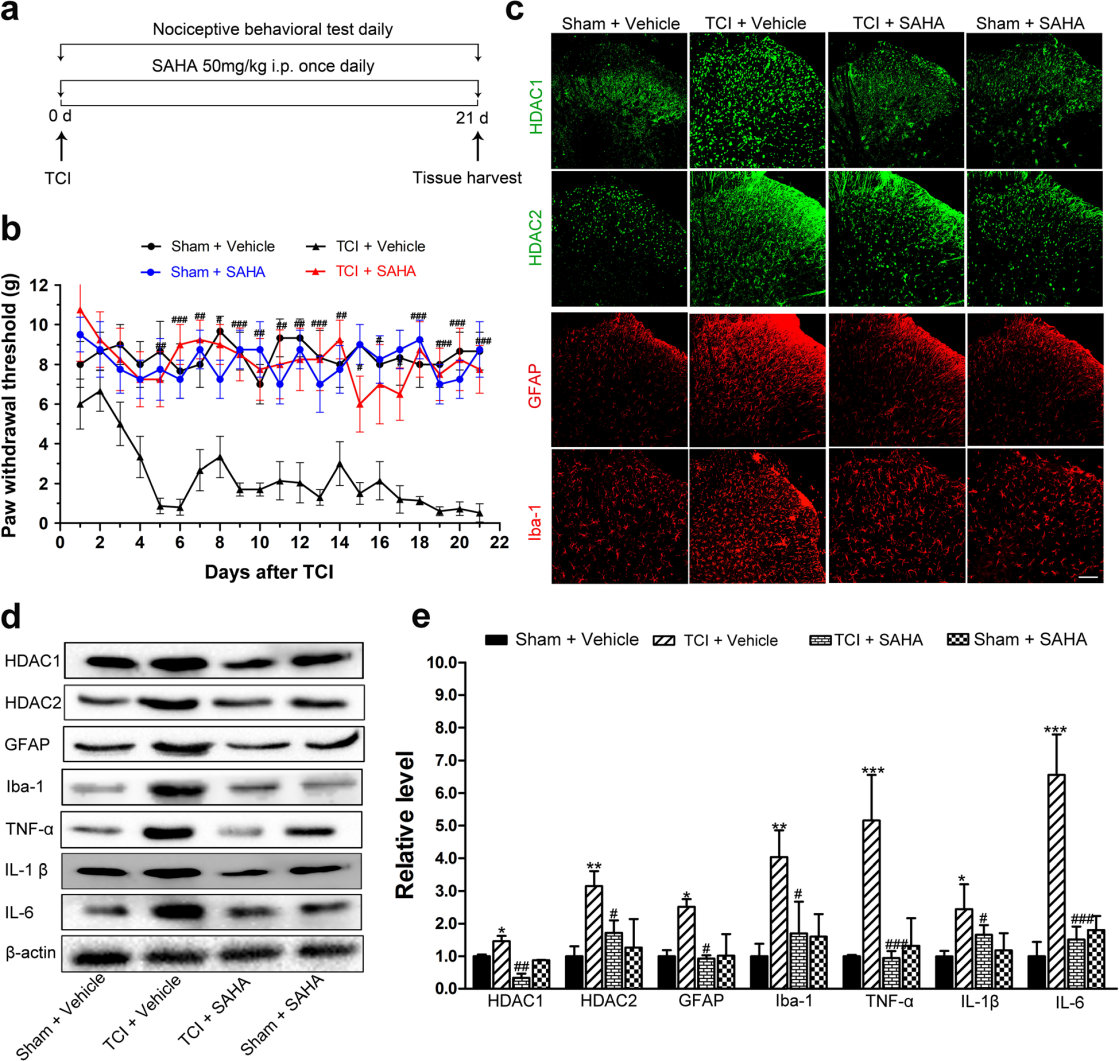
The effects of SAHA on TCI-induced mechanical allodynia and neuroinflammation in the spinal dorsal horn. **a** Experimental paradigms. **b** The effect of i.p. administration of SAHA on mechanical allodynia of TCI rats (*n* = 8). **c** Immunofluorescent staining of HDAC1 (green), HDAC2 (green), GFAP (red), and Iba-1 (red) in the spinal dorsal horn of the Sham + vehicle, the TCI + vehicle, the TCI + SAHA, and the Sham + SAHA group on POD 21. Scale bar = 100 μm. Representative bands (**d**) and quantitative analysis of HDAC1, HDAC2, GFAP, Iba-1, TNF-α, IL-1β, and IL-6 (**e**) in the spinal dorsal horn of Sham + vehicle, the TCI + vehicle, the TCI + SAHA, and the Sham + SAHA group. (*n* = 4). Analysis was based on the mean gray values and normalized to β-actin. Data are expressed as mean ± SEM. *p < 0.05, **p < 0.01 ***p < 0.001 versus the Sham + vehicle group, and ^#^*p* < 0.05, ^##^*p* < 0.01, ^###^*p* < 0.001 versus the TCI + vehicle group

To test whether inhibiting HDACs could ameliorate BCP, 50 mg/kg of SAHA, a widely and clinically used HDAC inhibitor, was i.p. injected daily for 21 consecutive days (Fig. 6a). Compared with the TCI + vehicle group, i.p. administration of SAHA significantly elevated the PWTs of the TCI rats, the effect of which persisted from POD 5 to POD 21 (Fig. 6b). In addition, there was no difference in the PWTs between the Sham + vehicle and the Sham + SAHA rats (Fig. 6b).

The immunofluorescent staining and Western blot analysis showed that i.p. administration of SAHA significantly inhibited the TCI-induced upregulation of HDAC1, HDAC2, GFAP, and Iba-1 in the spinal dorsal horn on POD 21 (Fig. 6c–e). However, their expression levels in the Sham rats were not obviously influenced by SAHA treatment (Fig. 6c–e). Consistently, Western blot analysis also showed that the expression levels of TNF-α, IL-1β, and IL-6 in the spinal dorsal horn of the TCI + SAHA rats were significantly lower than those of the TCI + vehicle rats (Fig. 6d, e). Meanwhile, there were no significant differences in these expression levels of proinflammatory cytokines between the Sham + vehicle and the Sham + SAHA groups (Fig. 6d, e). Collectively, these results indicated that SAHA treatment could mitigate TCI-induced neuroinflammation in the spinal dorsal horn, which may be involved in attenuating mechanical allodynia.

To exclude the possibility that SAHA may work through outside of spinal dorsal horn and dorsal root ganglia, the analgesic effects of i.t. administrated SAHA on BCP were further analyzed. Consistently, i.t. administrated SAHA significantly elevated the PWTs of the TCI rats from POD 6 to POD 14 (Additional file 2: Figure S2 a, b). Meanwhile, Western blot analysis showed that i.t. administration of SAHA could reverse TCI-induced upregulation of HDAC1 and 2 in the spinal dorsal horn (Additional file 2: Figure S2 c, d).

### The effects of SAHA on satellite glial cell-mediated neuroinflammation induced by TCI in the dorsal root ganglia

**Fig. 7:**
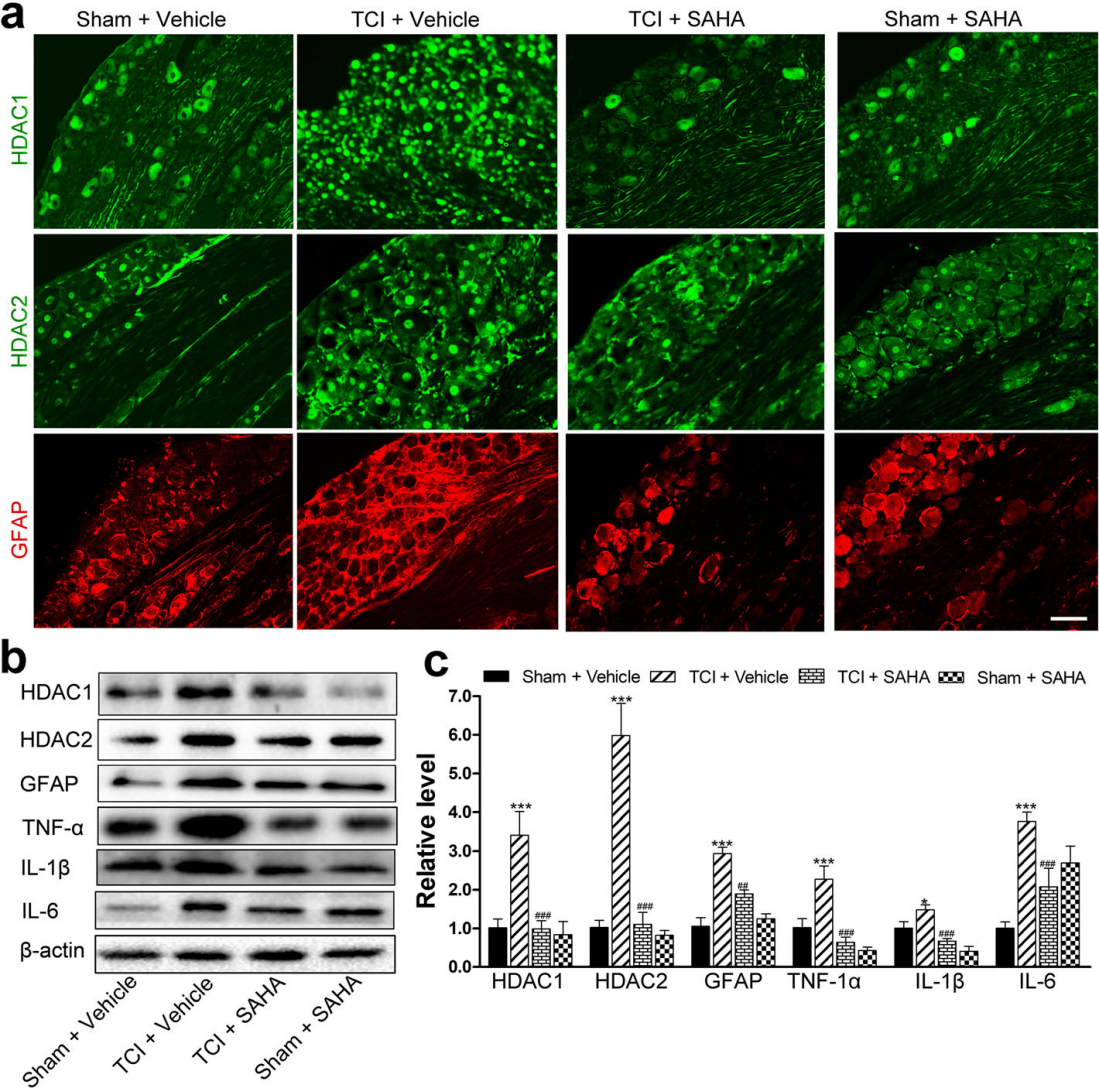
The effects of SAHA on TCI-induced neuroinflammation in the dorsal root ganglia. **a** Immunofluorescent staining of HDAC1 (green), HDAC2 (green), and GFAP (red) in the dorsal root ganglia of the Sham + vehicle, the TCI + vehicle, the TCI + SAHA, and the Sham + SAHA group on POD 21. Scale bar = 100 μm. Representative bands (**b**) and quantitative analysis of HDAC1, HDAC2, GFAP, TNF-α, IL-1β and IL-6 (**c**) in the dorsal root ganglia of Sham + vehicle, the TCI + vehicle, the TCI + SAHA and the Sham + SAHA group (*n* = 4). Analysis was based on the mean gray values and normalized to β-actin. Data are expressed as mean ± SEM. *p < 0.05 and ***p < 0.001 versus the Sham + vehicle group, and  ^##^*p* < 0.01, ^###^*p* < 0.001 versus the TCI + vehicle group

Accordingly, both the immunofluorescent staining and Western blot analysis showed that i.p. administration of SAHA could significantly inhibit the increased expression of HDAC1, HDAC2 and GFAP in the dorsal root ganglia on POD 21 (Fig. 7a–c). However, their expression levels in the Sham rats were not obviously influenced by SAHA treatment (Fig. 7a–c). Consistently, the Western blot analysis showed that i.t. administration of SAHA reversed TCI-induced upregulation of HDAC1 and 2 in the dorsal root ganglia (Additional file 2: Figure S2 e, f). After SAHA administration, the expression levels of TNF-α, IL-1β, and IL-6 in the dorsal root ganglia were significantly lower than those of the TCI + vehicle rats (Fig. 7b, c) with regard to Western blot analysis. There were no significant differences of these expressions between the Sham + vehicle and the Sham + SAHA group (Fig. 7b, c).

### SAHA administration could not alleviate TCI-induced bone destruction

**Fig. 8:**
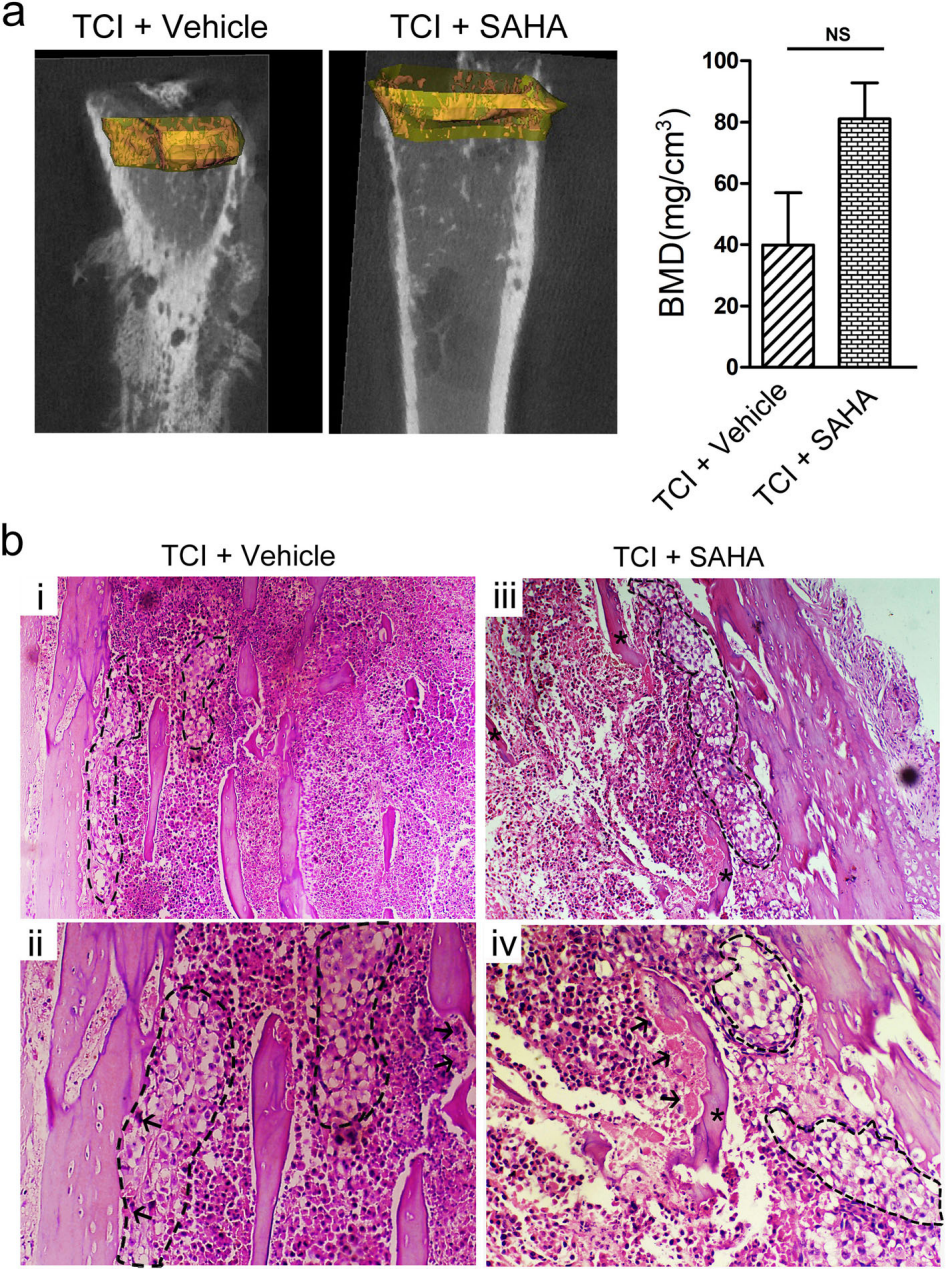
The effect of SAHA administration on bone destruction following TCI. **a** 2D representative MicroCT images of trabecular bone microarchitecture. **b** Quantitative analysis of the BMD in tibia of the TCI + vehicle and the TCI + SAHA group (*n* = 4). Data are expressed as the mean ± SEM. Representative images of HE staining showing cancer cell infiltration (cells within the dotted lines) and bone resorption pits (arrows) on trabecular surface in tibial marrow cavity of the TCI + vehicle (**b** (i, ii)) and TCI + SAHA (**b** (ii, iii)) group. Original magnification: 100 (upper row), 200 (bottom row)

To investigate whether the analgesic effects of SAHA were derived from its anti-tumor effects, we evaluated cancer growth and cancer-induced bone destruction in the tibia after SAHA administration on POD 21. MicroCT scanning displayed radiolucent lesions in the epiphysis and erosion of the cortical bone in the tibias of the TCI + vehicle and the TCI + SAHA group on POD 21. The continuity of the periosteum and the cortical bone broke off in several cases in the tibia of the TCI + vehicle and the TCI + SAHA. However, no obvious radiolucent lesions were observed in the tibia of the Sham + SAHA group (Fig. 8a). Quantitative analysis from microCT scanning showed that there was no significant difference between the TCI + vehicle and the TCI + SAHA group (Fig. 8a). Similarly, HE staining showed severe cancer cell infiltration, with much osteoclastic resorption pits attached on the trabecular surfaces in the tibial marrow cavity of the TCI + SAHA and TCI + vehicle groups (Fig. 8b).

### The effects of SAHA administration on GSK3β activities in the spinal dorsal horn and dorsal root ganglia following TCI

**Fig. 9:**
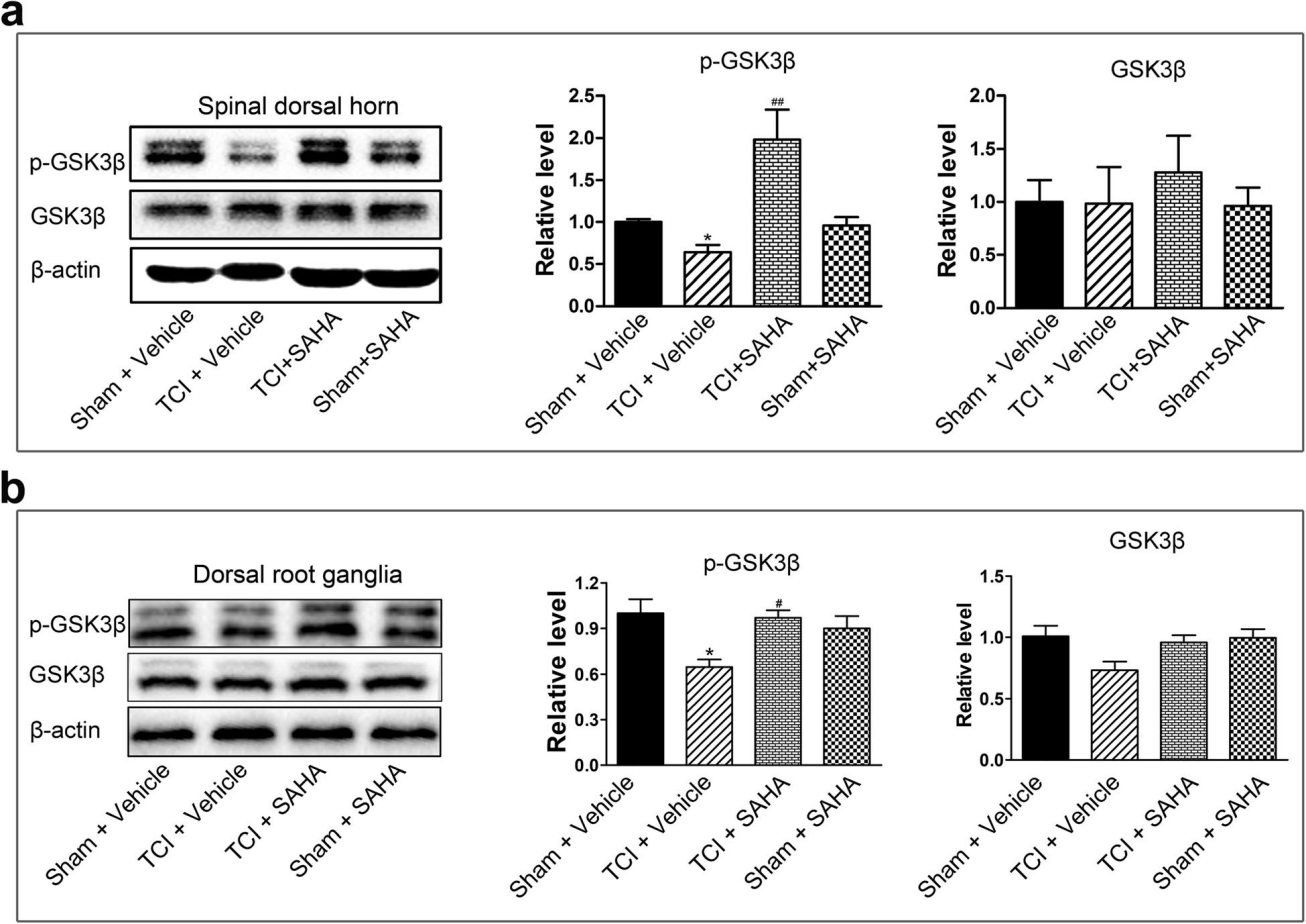
The inhibitory effects of SAHA on GSK3β activities in the spinal dorsal horn and dorsal root ganglia on POD 21. **a** Representative bands and quantitative analysis of p-GSK3β and GSK3β in the spinal dorsal horn of the Sham + vehicle, the TCI + vehicle, the TCI + SAHA and the Sham + SAHA group (*n* = 4). Analysis was based on the mean gray values and normalized to β-actin. **b** Representative bands and quantitative analysis of p-GSK3β and GSK3β in the dorsal root ganglia of the Sham + vehicle, the TCI + vehicle, the TCI + SAHA and the Sham + SAHA group (*n* = 4). Analysis was based on the mean gray values and normalized to β-actin. Data are expressed as the mean ± SEM. *p < 0.05 versus the Sham + vehicle group and ^#^*p* < 0.05, ^##^*p* < 0.01 versus the TCI + vehicle group

Given that glycogen synthase kinase-3 beta (GSK3β) is a key point of convergence of many signaling pathways that modify neuroinflammation, we further explored whether SAHA administration alleviate BCP by inhibiting GSK3β activities. The Western blot analysis showed that the expression levels of p-GSK3β (at serine 9, inactive form of GSK3β) in the spinal dorsal horn and dorsal root ganglia significantly decreased on POD 21 following TCI. When SAHA was i.p. injected, the expression levels of p-GSK3β in the spinal dorsal horn and dorsal root ganglia increased significantly. In addition, the expression levels of total GSK3β were not affected by SAHA administration (Fig. 9a, b).

**Fig. 10:**
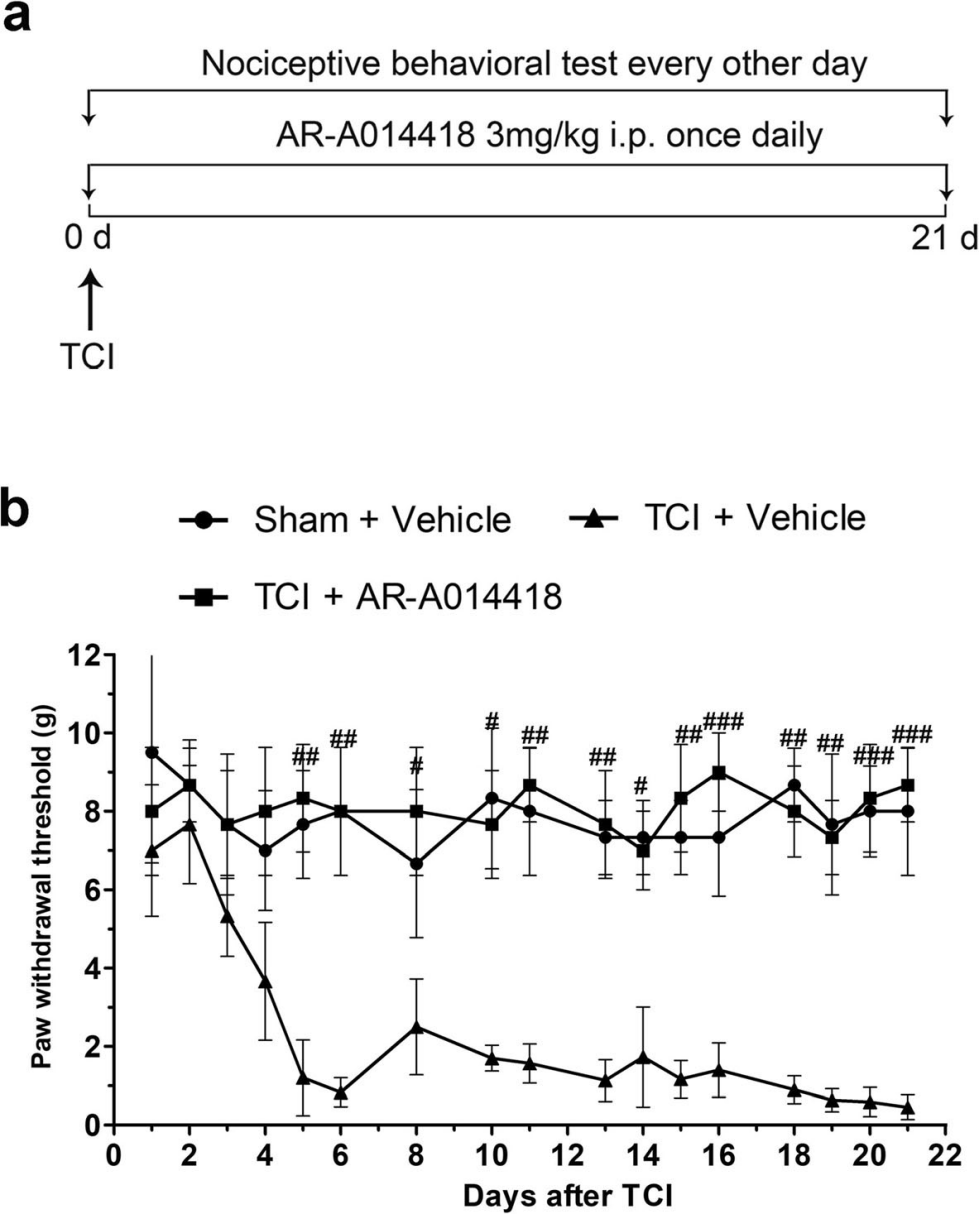
The anelgesic effects of AR-A014418 on TCI-induced mechanical allodynia. **a** Experimental paradigms. **b** The effect of i.p. administration of AR-A014418 on mechanical allodynia of TCI rats (*n* = 8). Data are expressed as mean ± SEM. ^#^*p* < 0.05, ^##^*p* < 0.01, ^###^*p* < 0.001 versus the TCI + vehicle group

To further confirm whether inhibiting GSK3β activity could attenuate mechanical allodynia in TCI rats, AR-A014418 (3 mg/kg, i.p.) was used to block GSK3β activity. Compared with the TCI + vehicle group, i.p. AR-A014418 treatment significantly elevated the PWTs of the ipsilateral hind paws, the effects of which started on POD 5 and lasted until the final detection day (Fig. 10a, b). Given that i.p. administrated AR-A014418 could contribute to pain relief by inhibiting GSK3β activity outside of the spinal dorsal horn and the dorsal root ganglia, the analgesic effects of i.t. administrated AR-A014418 on BCP were further analyzed. Consistently, the nociceptive behavioral tests showed that i.t. administrated AR-A014418 could significantly elevate the PWTs of the TCI rats from POD 6 to POD 14 (Additional file 3: Figure S3 a, b).

## Discussion

In the present study, we found that the expression levels of HDAC1 and HDAC2 in glial cells of the spinal dorsal horn increased sharply in the rat model of BCP, which was quite different from their distributions in the SNL rats. Meanwhile, our data also showed that HDAC1 and HDAC2 in the satellite glial cells of dorsal root ganglia increased persistently following TCI. Inhibition of HDACs by SAHA, a clinically used HDAC inhibitor, could significantly alleviate BCP, reverse TCI-induced upregulation of HDACs, and inhibit the activation of glial cells and accompanying neuroinflammation in the spinal dorsal horn and dorsal root ganglia. We also confirmed that the analgesic effects of SAHA were not due to its anti-tumor effects. Moreover, we found that the administration of SAHA could significantly upregulate the expression levels of p-GSK3β in the spinal dorsal horn and dorsal root ganglia to inhibit GSK3β activities, which may exert analgesic effects on the BCP. Collectively, these findings suggest that suppression of HDACs by SAHA administration may exert analgesic effects on BCP via inhibiting glia cell-mediated neuroinflammation in the spinal dorsal horn and dorsal root ganglia.

When cancer cells metastasize in bone, both cancer cells and their associated stromal cells generate pain by releasing algogenic substances, such as protons and bradykinin [27, 28]. These released factors can induce sensitization and activation of the nerve fibers that innervate the bone. Moreover, the released factors can stimulate a remarkable activation of osteoclasts, which cause severe bone destruction [29, 30]. Consistent with previous studies [27–30], our data showed severe cancer infiltration and osteoclast activation in the tibial marrow cavity following TCI (Fig. 1a, b). The PWTs of the ipsilateral hind paws to Von Frey hairs stimulation showed a continual and dramatic reduction (Fig. 1c). These results indicate that TCI caused marked bone destruction and lasting mechanical allodynia in the hind paws.

An epigenetic point of view of pain may reveal a new paradigm for developing pain management strategies [31, 32]. It has been reported that HDACs in the spinal dorsal horn are implicated in nociceptive sensitization of neuropathic and inflammatory pain [19, 33]. Emerging evidence have also demonstrated that the expression levels of HDAC1 and HDAC2 in the spinal dorsal horn and dorsal root ganglia increased continually following TCI, which may contribute to the downregulation of MOR in the spinal dorsal horn and dorsal root ganglia [24, 34, 35]. Hence, to confirm the involvement of HDACs in central sensitization of BCP, we firstly screened the expression levels of HDACs (HDAC1~HDAC6) in the spinal dorsal horn at different time points following TCI. Our data showed that the expression levels of HDACs (HDAC1, HDAC2 and HDAC4) in TCI and Sham rats were quite different (Fig. 2a–c, e). When the PWTs of the TCI rats decreased persistently, the expression levels of HDAC1 and HDAC2 in the spinal dorsal horn increased in a time-dependent manner with regard to Western blot and qRT-RCR analysis (Fig. 2b, c and Additional file 1: Figure S1). Thus, we speculated that the gradual increment of HDACs is associated with the degree of pain. The upregulation of HDAC1 and HDAC2 in the spinal dorsal horn following TCI was also consistent with previous reported research [35], suggesting that HDAC1 and HDAC2 may be among the most pivotal HDACs in the modulation of pain states. Moreover, we found the expression levels of HDAC4 in the spinal dorsal horn of the TCI rats were decreased significantly from POD 14; however, the underlying mechanism requires further research (Fig. 2e). In addition, although the expression levels of HDAC3, HDAC5, and HDAC6 in the spinal dorsal horn were not changed following TCI (Fig. 2d, f, g), it does not mean that they are not important in pain management during BCP, since HDACs can be activated by changing their distribution sites in pathological conditions [36]. The increased expression levels of HDACs in the dorsal root ganglia have been reported in our previously published research [24], hence we did not explor TCI-induced alterations of HDACs in the dorsal root ganglia in the present study.

Accumulative evidence has indicated that HDACs played important roles in central sensitization of BCP, and the underlying mechanisms mainly focus on HDAC-mediated effects on neuroplasticity. For example, Hou’s and our previous researches have both indicated that HDACi could restore MOR or KCC expressions in a rat [34, 35]. However, it should be noted that the activation of glial cells in the spinal dorsal horn and dorsal root ganglia also play proactive roles in the pathogenesis of chronic BCP, and the modulation of the activated glial cells could be a potential target for pain relief [36, 37]. Given that inhibition of HDACs is a promising molecular switch to epigenetically modify microglia behavior and astrocyte neurotoxicity from proinflammatory to anti-inflammatory, which could mitigate glia-mediated neuroinflammation in vitro and in vivo [17, 38, 39], we speculated that HDACs might also contribute to the activation of glial cells in the spinal dorsal horn and dorsal root ganglia during the development of BCP. As expected, we found that HDAC1 in spinal microglia and neurons, and HDAC2 in spinal astrocytes increased sharply and continually following TCI (Figs. 3 and 4). Meanwhile, the HDAC1 and HDAC2 in the satellite glial cells of the dorsal root ganglia also increased in a time-dependent manner following TCI (Fig. 5a, b). Moreover, we found that the HDAC1 in the dorsal root ganglia could transport from cytoplasm to nuclei following TCI. The cytoplasm-nuclei translocation of HDAC1 was consistent with our previous published researches [24], but the underlying mechanism still requires further research.

It has been reported that glial activation can be observed in both BCP and SNL, but glial cells are activated more robustly in the rat model of BCP than in the rat model of SNL [10, 12]. In the present study, rats with SNL (Sham and POD 14) were included in the present study to identify different roles of HDACs in rat model of BCP and neuropathic pain. We found that although both the TCI and SNL could lead to upregulation of HDAC1 and HDAC2 in the spinal dorsal horn, the distribution sites of increased HDAC1 and HDAC2 was quite different. The elevated spinal HDAC1 was mainly located in neurons and microglia following TCI, while it was mainly located in neurons following SNL. As for HDAC2, the upregulated spinal HDAC2 was mainly located in astrocytes in the TCI group, while it was mainly located in spinal neurons in the SNL group (Fig. 3 and 4). These results implied that the upregulation of HDAC1 and HDAC2 in the spinal dorsal horn of BCP and neuropathic pain was quite different, and closely related with their unique neurochemical changes. The reasons for the different neurochemical changes between BCP and neuropathic pain have been addressed in several researches [27, 40, 41]. The unique innervation pattern of skeletal structure and the acidic microenvironment generated by osteoclasts and cancer cells may both contribute to the different neurochemical changes of BCP and neuropathic pain [27, 42]. Previous studies have declared that nerve injury or noxious can induce the changes of HDACs in the spinal dorsal horn and dorsal root ganglia [20, 43]. Thus, we hypothesized that noxious stimulus generated by cancer and osteoclast cells might activate the HDACs in glial cells and neurons through the unique innervation pattern of skeletal structure, which was quite different from the activation of HDACs in neuropathic pain. Overall, our data implied that besides their roles in neuroplasticity, HDACs (HDAC1 and HDAC2) might also contribute to the particular activation of glial cells in spinal dorsal horn following TCI.

Emerging evidences have indicated that HDACi administration can exert analgesic effects on neuropathic pain and inflammatory pain [8, 9, 20, 44]. Denk and coworkers have indicated that long-term preventive administration of HDACi, such as MS-275, before any insult can reduce mechanical and thermal sensitivity of neuropathic pain but cannot affect already established neuropathic pain, which suggest that HDACs might be involved in the emergence of hypersensitivity during chronic pain [9]. Chiechio has demonstrated that intraperitoneally injected HDACi (TSA or SAHA) can reduce the second phase (approximately 20–45 min after formalin injection) of inflammatory pain [20]. In the present study, SAHA administration (i.p., 50 mg/kg) began at the same day when TCI operation was performed. Our data showed that the mechanical allodynia in the ipsilateral hind paws of TCI was attenuated by repetitive SAHA injections (Fig. 6a, b). The reversal of pain states by HDACi administration in the rat model of BCP agrees with our previous reported research and other’s [24, 34], but quite different from its preventive effects on neuropathic pain as reported before [20]. The controversy might lie in several reasons: (1) the roles of HDACs in neuropathic pain and BCP are quite different; our data showed that the upregulation and distribution of HDACs in the rat model of BCP were quite different from those of neuropathic pain (induced by SNL); (2) the different types and doses of HDACi used in these two studies, MS-275 (30 nmol/d, i.t.,) was used to attenuate neuropathic pain in a prior study [9], while SAHA (i.p., 50 mg/kg) was applied to attenuate BCP in the present study.

The effects of HDACi on the activation of microglia and astrocytes still remain controversial. Some studies have demonstrated that HDACi can induce anti-inflammatory activities via the suppression of microglial activation [16, 45–47]. Our previous research has also implied that triptolide can block the glial cell-mediated neuroinflammation [23]. However, a few studies have shown that HDACi significantly enhances the release of prostaglandins in LPS-activated microglia [13]. In the present study, we found that SAHA administration significantly decreased TCI-induced upregulation of HDAC1 and HDAC2 in the spinal dorsal horn and dorsal root ganglia, and largely diminished the activation levels of microglia and astrocytes in the spinal dorsal horn, and satellite glial cells in the dorsal root ganglia (Figs. 6c–e and 7a–c). We also found that SAHA administration could not affect basal HDACs of the Sham group (Fig. 6c–e), which agreed with previously published researches [48]. Other studies have also indicated that SAHA can selectively alters the transcription of relatively few genes, and normal cells are at least ten fold more resistant than transformed cells to SAHA and related HDAC inhibitor-induced cell death (for example, see [49]). In this regard, SAHA is generally acceptable for primary-level research. Moreover, the expression levels of proinflammatory cytokines, such as TNF-α, IL-1β, and IL-6, in the spinal dorsal horn and dorsal root ganglia of TCI rats recovered after SAHA administration (Figs. 6d, e and 7b, c). Altogether, these results indicated that inhibiting HDACs by SAHA could inhibit the activation of glial cells and accompanying production of proinflammatory cytokines in the spinal dorsal horn and dorsal root ganglia during the pathogenesis of BCP.

Though it is irrefutable that i.p. administrated SAHA not only reversed TCI-induced HDAC upregulation in SDH and DRG but also inhibited the activation of glial cells, but it still exist the possibility that SAHA exerts its role in pain relieving through inhibiting HDACs outside of SDH and DRG, then inhibits glial activation and HDAC expression due to pain relieving. To rule out this possibility, i.t. injection with SAHA was especially performed. Consistently, our data showed that i.t. administrated SAHA could not only attenuate the mechanical allodynia of the ipsilateral hind paws of TCI but also strongly reverse TCI-induced upregulation of HDACs in the dorsal horn and dorsal root ganglia. These finding strongly indicated that the analgesia of SAHA was derived from its inhibitory effects on glial activation in the spinal dorsal horn and dorsal root ganglia.

SAHA was licensed in 2006 for the treatment of cutaneous T cell lymphoma following the Food and Drug Administration approval [50]. Although SAHA has been shown to be effective against a defined subset of hematologic tumors, there is less than convincing evidence that SAHA will be effective against tumors as single agents [51]. Actually, SAHA is usually used to enhance the cytotoxicity of other anti-cancer drugs [50, 52, 53]. It has been reported that SAHA can sensitize breast cancer cells to apoptosis induced by a series of anti-tumor agents, such as MD5–1 [54], olaparib [55] and parthenolide [56], while SAHA alone cannot generate these effects [57]. In the current study, we investigated the effects of SAHA administration on bone destruction of the tibia in the TCI + SAHA group. The HE staining showed that infiltration of cancer cells and severe osteolytic lesions could be easily observed in the proximal epiphysis of the tibias of the TCI + SAHA rats (Fig. 8a, b). The microCT scanning also showed large radiolucent lesions in the bone marrow cavity and severe erosion of the cortical bone in the tibias of the TCI + SAHA rats (Fig. 8a). These results suggested that SAHA administration could not inhibit cancer growth or cancer-induced bone destruction, thus the analgesic effects of SAHA on BCP are not derived from its anti-tumor effects.

Glycogen synthase kinase-3 beta (GSK3β) has been considered as a crucial regulator of the balance between pro- and anti-inflammatory responses in both the peripheral and central nervous systems [58]. It has been reported that GSK3β activity in the spinal dorsal horn increases during the late stage of neuropathic pain and that suppressing GSK3β activity can significantly ameliorate mechanical allodynia [59]. Martins and coworkers also showed that GSK3β inhibitors could produce anti-hyperalgesic effects and decrease proinflammatory cytokines, such as TNF-α and IL-1β [60]. Moreover, Dobashi et al. demonstrated that Valproate, another HDAC inhibitor, could attenuate the development of morphine tolerance by inhibiting GSK3β activity [61]. Considering (i) the critical role of microglial and astrocytic activation in a rat model of BCP, (ii) the significant roles of GSK3β in neuroinflammation, and (iii) the neuroprotective and anti-inflammatory properties of HDACi via the GSK3β pathway, we further postulated that GSK3β activity might be involved in HDAC-mediated activation of glial cells during the pathogenesis of BCP. Consistent with previous research [59, 61, 62], our data showed that GSK3β activity increased on POD 21, while i.p. administration of SAHA significantly reduced GSK3β activities in spinal dorsal horn and dorsal root ganglia (Fig. 9a–f). Suppression of GSK3β activity by AR-A014418 administration, a thiazole and an ATP competitive inhibitor of GSK3β [63], significantly relieved mechanical allodynia in TCI rats, indicating a close correlation of BCP and GSK3β activity (Fig. 8g, h and Additional file 3: Figure S3 a, b). These results supported the conclusion that SAHA might relieve BCP by suppressing GSK3β activities in the spinal dorsal horn and dorsal root ganglia. However, it should be noted that our data could not indicate that the upregulation of p-GSK3β was due to the inhibition of HDAC1/HDAC2, since SAHA administration could inhibit all 11 known human class I and class II HDACs (HDAC3-HDAC11) [53]. In our future research, we will compare the different effects of SAHA and GSK3β inhibitors on activation of glial cells between intrathecal administration and intraperitoneal administration, and specific designed inhibitor, such as siRNA, would be used to interrogate the modulatory effects of HDAC1/HDAC2 on activities of GSK3β.

## Conclusion

Collectively, our data suggested that the upregulation of HDAC1 and HDAC2 was implicated in the over-activation of glial cells in the spinal dorsal horn and dorsal root ganglia during pathogenesis of BCP. Inhibition of HDACs by SAHA reversed the glial activation and relieved pain behavior following TCI. Meanwhile, the analgesic effects of SAHA on BCP were not due to its anti-tumor effects. Moreover, the activity of GSK3β might be a potential target regulated by HDACs that participates in pain management during BCP. Our findings indicate that HDACs may be involved in development of BCP via glia-mediated neuroinflammation and suggest the inhibition of HDACs as a novel strategy for treating BCP.

## Supplementary information

**Additional file 1 **Figure S1. Gene expression of *HDAC1~HDAC6* in the spinal dorsal horn at various time points (sham, POD 7, POD 14 and POD 21) following TCI. Relative mRNA expression levels of *HDAC1* (a), *HDAC2* (b), *HDAC3* (c), *HDAC4* (d), *HDAC5* (e) and *HDAC6* (F) in the spinal dorsal horn of TCI rats (*n* = 3). Data are expressed as the mean ± SEM. *p < 0.05, and ***p < 0.001 versus the Sham group.

**Additional file 2 **Figure S2. The effects of i.t. administrated SAHA on TCI-induced mechanical allodynia and upregulation of HDACs. (a) Experimental paradigms. (b) The effects of i.t. administrated SAHA on mechanical allodynia of TCI rats (*n* = 6 for each group). (c and d) Representative bandsand quantitative analysis of HDAC1 and HDAC2 in the spinal dorsal horn of the TCI + Vehicle and the TCI + SAHA group (*n* = 4). (e and f) Representative bands and quantitative analysis of HDAC1 and HDAC2 in the dorsal root ganglia of the TCI + Vehicle and the TCI + SAHA group (*n* = 4). Data are expressed as mean ± SEM. **p < 0.01 ***p < 0.001 versus the TCI + Vehicle group.

**Additional file 3 **Figure S3. The effects of i.t. administrated AR-A014418 on TCI-induced mechanical allodynia. (a) Experimental paradigms. (b) The effect of i.t. administration of AR-A014418 on mechanical allodynia of TCI rats (*n* = 6 for each group). Data are expressed as mean ± SEM. *p < 0.05, **p < 0.01 ***p < 0.001 versus the TCI + Vehicle group.

**Additional file 4.** Table S1. Primer sequences used in this study.

## Data Availability

All data generated or analyzed during this study are available from the corresponding author on reasonable requests.

## Abbreviations

BCP: Bone cancer pain
GFAP: Glial fibrillary acidic protein
GSK3: Glycogen synthase kinase-3
HDAC: Histone deacetylase
HDACi: Histone dedacetylase inhibitors
HE: Hematoxylin and eosin
Iba-1: Ionized calcium-binding adaptor molecule 1
IL: Interleukin
PBS: Phosphate-buffered saline
POD: Postoperative day
PWT: Paw withdrawal threshold
qRT-PCR: Quantitative real-time reverse transcriptional polymerase chain reaction
SAHA: Suberoylanilide hydroxamic acid
SNL: Spinal nerve ligation
TCI: Tumor cell inoculation
TNF-α: Tumor necrosis factor-α
