# Supplementary figures and images for: Suppression of histone deacetylases by SAHA relieves bone cancer pain in rats via inhibiting activation of glial cells in spinal dorsal horn and dorsal root ganglia
Source: J Neuroinflammation. 2020 Apr 22;17:125. doi: 10.1186/s12974-020-01740-5 (PMC7175547; doi:10.1186/s12974-020-01740-5)

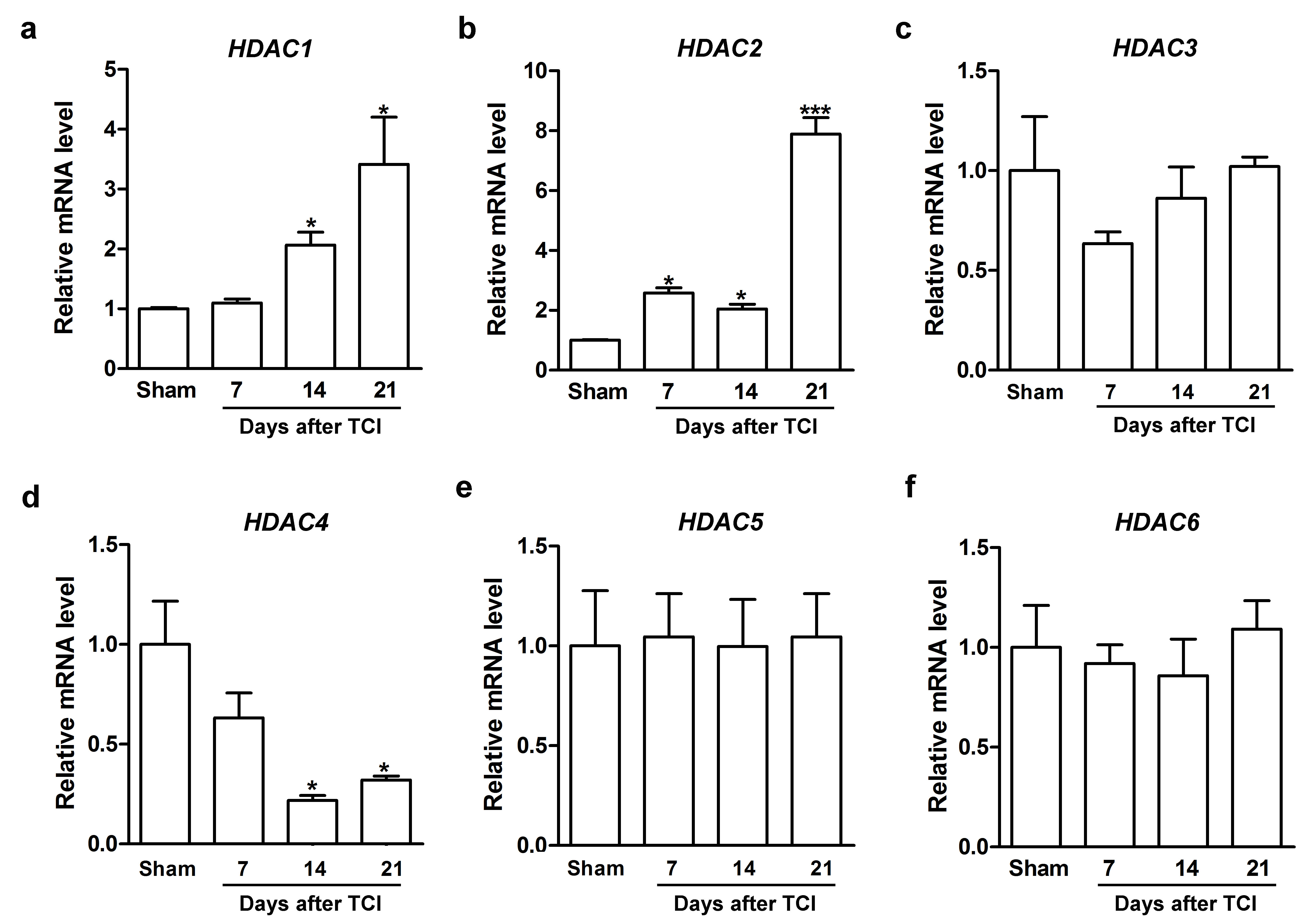

Supplement: Supplementary file 1 — Additional file 1 Figure S1. Gene expression of HDAC1~HDAC6 in the spinal dorsal horn at various time points (sham, POD 7, POD 14 and POD 21) following TCI. Relative mRNA expression levels of HDAC1 (a), HDAC2 (b), HDAC3 (c), HDAC4 (d), HDAC5 (e) and HDAC6 (F) in the spinal dorsal horn of TCI rats (n = 3). Data are expressed as the mean ± SEM. *p < 0.05, and ***p < 0.001 versus the Sham group. [file 12974_2020_1740_MOESM1_ESM.jpg]

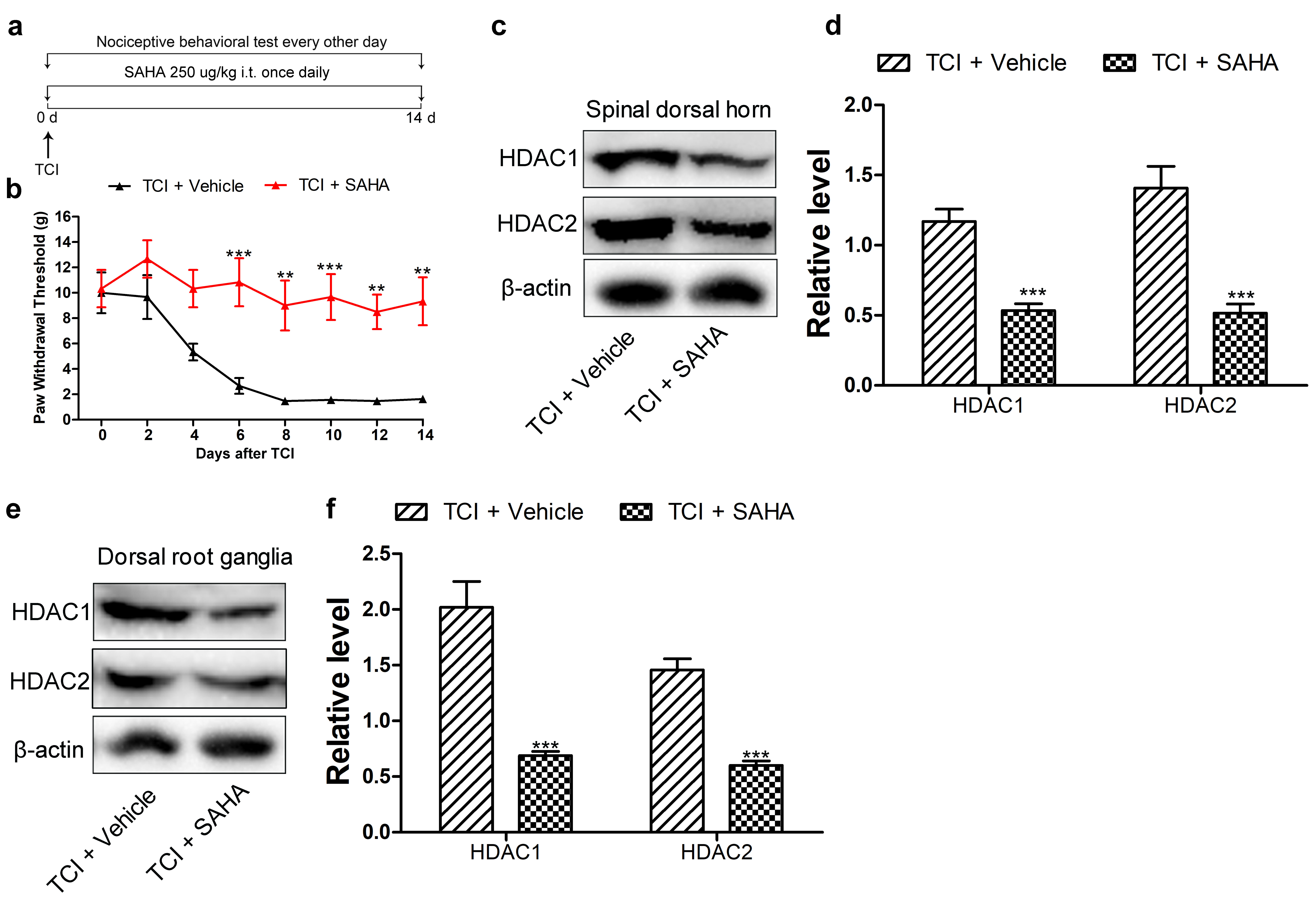

Supplement: Supplementary file 2 — Additional file 2 Figure S2. The effects of i.t. administrated SAHA on TCI-induced mechanical allodynia and upregulation of HDACs. (a) Experimental paradigms. (b) The effects of i.t. administrated SAHA on mechanical allodynia of TCI rats (n = 6 for each group). (c and d) Representative bandsand quantitative analysis of HDAC1 and HDAC2 in the spinal dorsal horn of the TCI + Vehicle and the TCI + SAHA group (n = 4). (e and f) Representative bands and quantitative analysis of HDAC1 and HDAC2 in the dorsal root ganglia of the TCI + Vehicle and the TCI + SAHA group (n = 4). Data are expressed as mean ± SEM. **p < 0.01 ***p < 0.001 versus the TCI + Vehicle group. [file 12974_2020_1740_MOESM2_ESM.jpg]

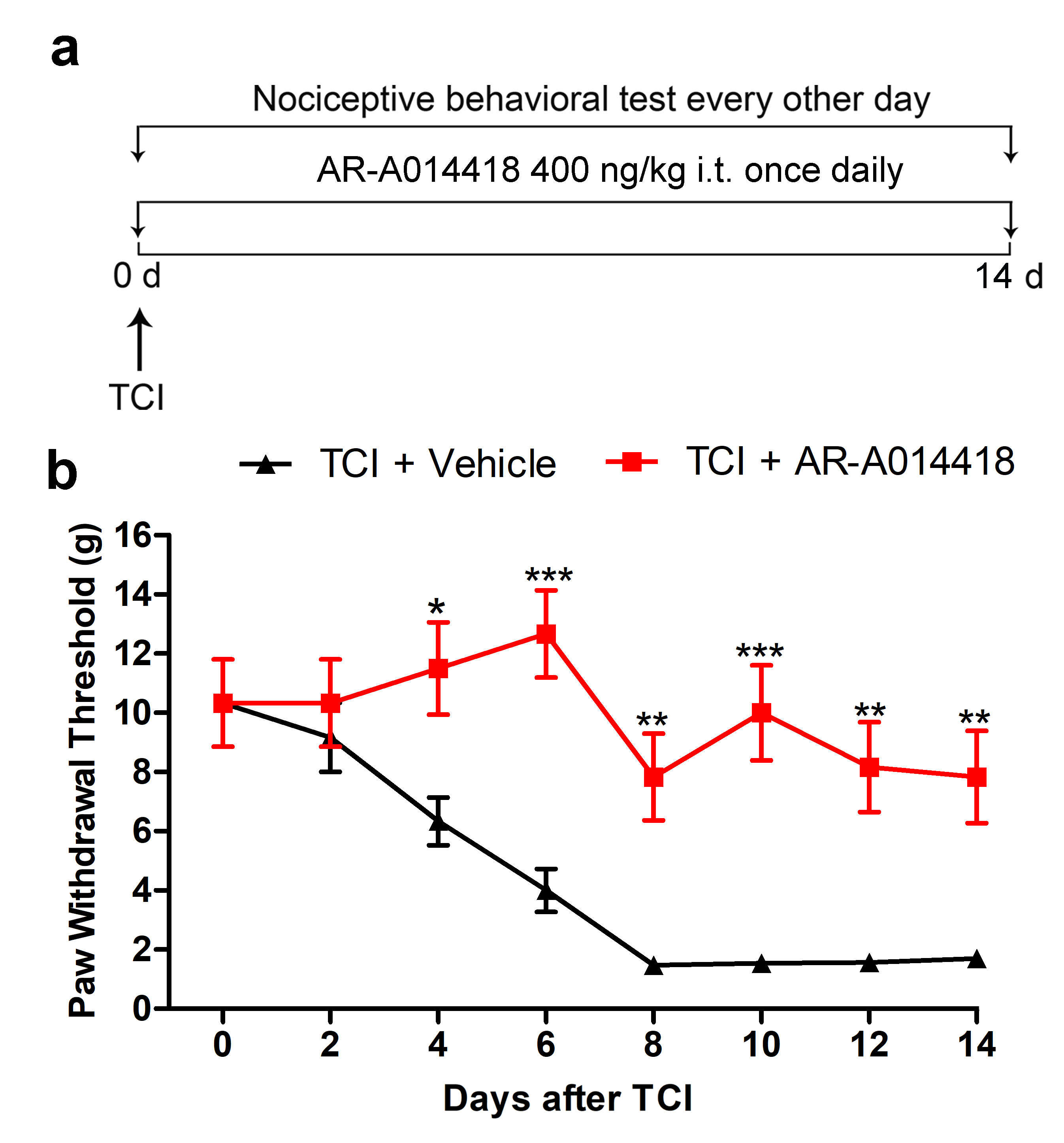

Supplement: Supplementary file 3 — Additional file 3 Figure S3. The effects of i.t. administrated AR-A014418 on TCI-induced mechanical allodynia. (a) Experimental paradigms. (b) The effect of i.t. administration of AR-A014418 on mechanical allodynia of TCI rats (n = 6 for each group). Data are expressed as mean ± SEM. *p < 0.05, **p < 0.01 ***p < 0.001 versus the TCI + Vehicle group. [file 12974_2020_1740_MOESM3_ESM.jpg]
