# Supplementary material for: Suppression of histone deacetylases by SAHA relieves bone cancer pain in rats via inhibiting activation of glial cells in spinal dorsal horn and dorsal root ganglia
Source: J Neuroinflammation. 2020 Apr 22;17:125. doi: 10.1186/s12974-020-01740-5 (PMC7175547; doi:10.1186/s12974-020-01740-5)
Supplement: Supplementary file 4 — Additional file 4. Table S1. Primer sequences used in this study. [file 12974_2020_1740_MOESM4_ESM.docx]

**Supplementary Table 1**. Primer sequences used in this study

| **Gene** | **Full name** | **Primer** | **Sequences (5’-3’)** |
| --- | --- | --- | --- |
| *HDAC1* | Histone deacetylase 1 | Forward | ACTGCGAGATGGCATTGATGA |
|  |  | Reverse | GAGTCTGAGCCGCACTGTAGGA |
| *HDAC2* | Histone deacetylase 2 | Forward | TGGGCTGCTTCAACCTAACTGTC |
|  |  | Reverse | ACTGCAGTCTCATACGTCCAACATC |
| *HDAC3* | Histone deacetylase 3 | Forward | CTGAACCATGCACCCAGTGTC |
|  |  | Reverse | CGGCATCCATGCTGCTCTTA |
| *HDAC4* | Histone deacetylase 4 | Forward | AAGCATGTGTTTCTGCTCTGCTG |
|  |  | Reverse | GCATTGGCATTGGGTCTCTG |
| *HDAC5* | Histone deacetylase 5 | Forward | CCGGCAGAAGCTGGACAGTAA |
|  |  | Reverse | CATTCGCACGGCACTAGAGG |
| *HDAC6* | Histone deacetylase 6 | Forward | TCTGGCGGACGAGAAAGAG |
|  |  | Reverse | GGGCGATTGGGGATTGT |
| *GAPDH* | Glyceraldehyde 3-phosphate dehydrogenase | Forward | CCATTCTTCCACCTTTGATGCTG |
|  |  | Reverse | GTCCAGGGTTTCTTACTCCTTGG |
